# Evaluating a Tailored Web-Based eHealth Intervention for Symptom Management in Couples Managing Prostate Cancer During the COVID-19 Pandemic: Randomized Clinical Trial

**DOI:** 10.2196/88717

**Published:** 2026-07-10

**Authors:** Lixin Song, Christine Rini, Yuexia Zhang, Chunxuan Ma, Laurel Northouse, Matthew E Nielsen, Xianming Tan, Ronald C Chen

**Affiliations:** 1 School of Nursing and Mays Cancer Center, The University of Texas at San Antonio, 7703 Floyd Curl Drive, San Antonio, TX, 78229, United States, 1 210 450 8561; 2 Department of Medical Social Sciences, Feinberg School of Medicine, Northwestern University, Chicago, IL, United States; 3 Department of Epidemiology and Biostatistics, Case Western Reserve University, Cleveland, OH, United States; 4 School of Nursing, University of Michigan, Ann Arbor, MI, United States; 5 Department of Biostatistics and the Lineberger Comprehensive Cancer Center, University of North Carolina at Chapel Hill, Chapel Hill, NC, United States; 6 Department of Radiation Oncology, University of Kansas Medical Center, Kansas City, KS, United States

**Keywords:** randomized controlled trial, prostate cancer, patient-partner dyads, quality of life, stress, coping, eHealth, COVID-19 pandemic, family-based research, RCT

## Abstract

**Background:**

Prostate cancer (PCa) is the most common nonskin cancer among men. Although treatments achieve excellent survival for localized PCa, long-lasting complications significantly diminish patients’ quality of life (QOL) across physical, sexual, psychosocial, and general symptom domains. Because these effects also profoundly impact intimate partners, often reducing partners’ QOL as much as or more than patients’ QOL, supportive care must address the needs of both members of the couple.

**Objective:**

This study evaluated the efficacy of the Prostate Cancer Education and Resources for Couples (PERC) eHealth intervention in improving outcomes for patients and their partners.

**Methods:**

We enrolled 280 dyads (560 individuals) consisting of patients with localized PCa who recently completed treatment and their partners through the North Carolina Cancer Registry. Dyads were randomized to PERC or a control group that received access to the National Cancer Institute prostate cancer website. PERC dyads completed a nurse-led orientation and received monthly follow-ups. The platform has the following three components: (1) 11 interactive modules with postsession assignments on QOL, symptom management, and cancer communication; (2) a moderated online forum providing professional and peer support; and (3) a resource toolbox containing scientific publications related to PCa care. PERC development was guided by an adapted stress-coping theory and informed by evidence and stakeholder input. Validated questionnaires assessed QOL (Functional Assessment of Chronic Illness Therapy–General [FACT-G] total; primary outcomes), FACT-G subdomains, and symptom and psychosocial measures (secondary outcomes) at baseline and at 4, 8, and 12 months. Multilevel linear mixed models tested intervention effects.

**Results:**

The trial was conducted during the COVID-19 pandemic. Among PERC (n=141) and control (n=139) dyads who completed baseline assessments, 221 (78.9%) dyads completed the 12-month follow-up (PERC=106 and control=115). FACT-G total score, subdomains, and overall psychosocial outcomes did not differ significantly between groups over time. Patients assigned to PERC reported better physical QOL (mean difference 0.9, 95% CI –0.1 to 1.9; Cohen* d*=0.33), less negative illness appraisal (mean difference 0.2, 95% CI 0.0-0.4; Cohen *d*=0.38), lower pain (mean difference –2.7, 95% CI –5.3 to –0.2; Cohen *d*=–0.38) at 12 months, and less frequent fatigue across time (mean difference –2.1, –95% CI –3.9 to –0.4; Cohen *d*=–0.23). PERC partners reported less urinary symptom bother at 8 months (mean difference 6.5, 95% CI –1.0 to 14.1; Cohen *d*=0.44).

**Conclusions:**

Although no significant between-group difference was observed in the FACT-G total score, PERC demonstrated exploratory benefits, including improved physical QOL, less fatigue, lower pain, and improved illness appraisal among patients, as well as less urinary bother among partners. These findings suggest that the COVID-19 pandemic may have adversely affected participants’ overall QOL, potentially obscuring changes in the primary outcome, while highlighting targeted benefits that warrant evaluation in larger studies.

## Introduction

Prostate cancer (PCa) is a significant health concern in the United States and other industrialized countries, ranking as the most prevalent nonskin cancer among men and the second leading cause of cancer-related deaths after lung cancer [1]. While treatments such as prostatectomy and radiation therapy offer promising 5-year survival rates of 97.9% for localized PCa, they often cause complications and adverse effects that impact patients’ quality of life (QOL) [2,3]. These effects, which may last for years, include urinary (eg, urinary urgency and incontinence) and bowel symptoms (eg, diarrhea), difficulties with sexual function (eg, erectile dysfunction) [4], psychosocial distress (eg, depression and anxiety) [5,6], as well as general symptoms such as pain, fatigue, and sleep disturbance [7].

For patients in intimate relationships, these adverse effects of PCa and its treatments also affect patients’ partners, who are critical sources of support throughout the survivorship journey. Referred to as a “couples’ illness” [8], PCa profoundly reduces partners’ QOL, sometimes to a greater extent than patients’ QOL [9]. Partners of men with PCa often experience anxiety, depression, and other somatic symptoms [10], mirroring the stress-coping challenges that patients face [11]. Given the interdependent relationship between patients living with PCa and their partners, addressing the supportive care needs of both is critical. Persistent supportive care needs among patients living with PCa and their partners, particularly in geographically dispersed populations, underscore the potential of accessible eHealth interventions to improve symptom management and QOL.

Our multidisciplinary team developed an eHealth intervention called Prostate Cancer Education and Resources for Couples (PERC) [12] to address the specific educational and symptom management needs of patients and their partners. Informed by evidence-based practice guidelines [13] and input from stakeholders, including survivors of PCa, partners (eg, a spouse or romantic partner), and oncology care providers [12], PERC aimed to enhance QOL by bolstering positive appraisals, self-efficacy, social support, and symptom management knowledge and skills. This study evaluated PERC’s efficacy on QOL as the primary outcome. Secondary outcomes were symptom management and stress coping-related psychosocial measures in patients living with PCa and their partners over time. We hypothesized that PERC users would report greater improvements in outcomes over time at 4, 8, and 12 months post baseline (T2-T4) when compared to attention control counterparts who received access to the National Cancer Institute’s (NCI) PCa website.

## Methods

### Study Design

This was a two-arm, parallel-group, longitudinal randomized controlled trial (RCT) (ClinicalTrials.gov NCT03489057). Implementation of the RCT, particularly follow-up data collection, coincided with the COVID-19 pandemic. This trial is reported according to the CONSORT (Consolidated Standards of Reporting Trials) statement (Checklist 1).

### Participants

Patients were eligible if they met the following criteria: (1) aged 40-75 years, (2) within 4 months after receiving radical prostatectomy or radiation therapy for localized PCa with curative intent, (3) had no other cancer history or ongoing treatment within the past 2 years, and (4) had an intimate partner willing to participate in the RCT. Exclusion criteria included a diagnosis of cancer or receipt of cancer treatment in the past calendar year. Both patients and partners had to be English speakers without cognitive impairment (based on <3 errors on the Short Portable Mental Status Questionnaire) [14].

### Procedure

After approval from the Institutional Research Board, we used the North Carolina Central Cancer Registry Rapid Case Ascertainment (RCA) service to identify and recruit participants, as we have described [12]. RCA, working with the state cancer registry, identified newly diagnosed patients living with PCa statewide as quickly as within 2 weeks of diagnosis. After sequentially sending physicians’ and patients’ opt-out letters, potential patient participants who did not opt out were screened for eligibility and provided consent for the research team to discuss participation with their partners. Each eligible patient and partner separately provided consent, forming a dyad for the study. Enrolled patients and partners independently completed the baseline survey (T1) and were randomly allocated as a dyad to either the PERC or control group. Our research nurse provided orientation and established rapport with participants in both groups, so they had continued access to support and engagement throughout the study. For participants without access to a computer or the internet, the study team provided a loaned iPad with a prepaid hotspot. To ensure equitable access to the program regardless of participants’ technology literacy, printed pamphlets with graphics and step-by-step illustrations for navigating the website were distributed via both mail and email. Additionally, technology orientation and ongoing support were offered so that participants were comfortable using the loaned iPads.

Follow-up surveys were conducted at 4, 8, and 12 months after baseline (T1), corresponding to T2, T3, and T4, respectively. All data were entered into a secure, Health Insurance Portability and Accountability Act–compliant Research Electronic Data Capture (REDCap) database (Vanderbilt University). Each participant received gift cards of US $20, US $30, US $30, and US $50 for completing the T1-T4 assessments, respectively.

### Randomization and Masking

After providing informed consent, patients and their partners independently completed the baseline survey (T1). They were then randomly assigned as dyads to either the PERC or the control group in a 1:1 ratio. Randomization was stratified by treatment type (prostatectomy or radiation therapy) to ensure balanced group allocation. Our team’s biostatistician (XT) implemented computer-generated permuted block randomization with variable block sizes, randomly selecting block sizes from the set (4, 6, and 8) to minimize predictability and maintain allocation concealment. This approach was applied within each stratum.

To maintain blinding, research assistants responsible for conducting surveys remained unaware of participants’ group assignments. Each participant received a unique username and password to access the study website. Upon logging in, participants were automatically directed to either the PERC or NCI PCa website based on their randomized group assignment.

### Attention Control Group

The NCI PCa website [15] is a publicly accessible resource offering comprehensive information on PCa treatment, research, causes, and statistics, along with general coping resources. It includes interactive features such as a toll-free phone number and LiveHelp online chat that provide support for cancer-related queries, clinical trials, and smoking cessation. As the RCT progressed, the NCI website continued to improve and expand its comprehensive library of resources for patients living with cancer and caregivers.

Our research nurse introduced the control dyads to the website in an orientation meeting in week 1 of the study, familiarizing them with its features and addressing any concerns or challenges they raised. Subsequently, dyads received monthly emails from the nurse interventionist to evaluate their symptoms, but the emails did not provide specific interventions to address their symptoms. Instead, participants were reminded to use the NCI website. Participants were encouraged to contact the research nurse with any questions about using the website.

### PERC Intervention

Guided by the adapted stress-coping theory [16], PERC harnessed eHealth technologies to enhance couples’ access to posttreatment supportive care at their convenience. Our research nurse engaged with PERC dyads at an orientation session in week 1 to familiarize them with the platform; in monthly follow-ups during weeks 2‐14 to evaluate their symptoms, monitor module completion, and help troubleshoot any problems that arose, if needed; and to encourage completion of a final wrap-up session in week 15. Participants could access the online platform at any time. As described in our protocol [12] and pilot study papers [17,18], the PERC platform had three key components:

1. Education modules: 11 modules with postsession assignments covered topics such as QOL improvement, symptom management (general and PCa-specific), and communication skills. Each module included patient-, partner-, and dyad-focused materials with interactive tasks to encourage mutual engagement. Evidence-based content was written at a sixth- to eighth-grade reading level and incorporated text, graphics, audio, and video to support patients and partners with diverse learning needs and educational backgrounds. The use of multiple media formats enhanced the learning experience.
2. Moderated online forum: this feature offered professional support from our trained nurse interventionist, who addressed symptom-related questions. It also provided a platform for dyads to seek guidance and share experiences within a supportive community of peer patients and caregivers navigating similar challenges with PCa and its treatment.
3. Resource toolbox: relevant scientific publications were available to offer additional sources of information and support for users seeking further insights into PCa care.

Overall, the PERC program was designed to enhance accessibility to supportive care for individuals with varying levels of health and technology literacy. The program used plain language, short modules, supportive graphics, and multiple multimedia formats (text, audio, and video) to facilitate comprehension and engagement. The platform also featured simplified navigation and guided support, including printed step-by-step instructions for using the site, a tutorial video, and online technical assistance. In addition, participants had access to the research nurse and technical support via phone, email, or mail when needed. These design features were tested in prior pilot feasibility studies [17,18], which received positive feedback regarding usability and accessibility. The version used in this trial was further refined to strengthen these features and support equitable access for participants with lower health or technology literacy.

### Measurements

Patients and partners independently completed validated, psychometrically sound questionnaires assessing outcomes (QOL, symptoms, and stress-coping variables) via phone or online, based on their preferences. The patient and partner versions of most questionnaires (unless otherwise indicated in the Measurements subheading) were similar, with slight wording adjustments to reflect assessment of their different roles.

### Primary Outcomes: QOL

Patients and partners separately reported their own QOL by completing the 27-item patient or partner version of the Functional Assessment of Chronic Illness Therapy-General (FACT-G) scale, respectively [19,20]. Both versions of the FACT-G include physical, emotional, social, and functional well-being subscales. The FACT-G total scores for patients and partners—the summation of the 4 subscale scores—were the primary outcomes.

### Secondary Outcomes

#### Overview

The FACT-G subdomain scores, defined as the sums of the items in the individual physical, emotional, social, and functional well-being subscales, were treated as secondary outcomes.

#### Psychosocial Outcomes

Illness appraisal was assessed by asking patients and partners to report their perceptions of illness and caregiving, respectively, using an adapted 20-item, 5-point Likert response Appraisal Scale [21], for example, “I feel things are going to get worse for me.”

Coping resources included self-efficacy—an individual’s confidence in managing PCa and related symptoms and stress—which was assessed using a 9-item, 10-point Likert scale adapted from the Lewis Cancer Self-Efficacy Scale [22].

Social support from people other than the intimate partner was assessed using the 8-item Patient-Reported Outcomes Measurement Information System (PROMIS) emotional [23], informational [24], and instrumental support [25] measures, which assess respondents’ perceptions that a particular type of support is available if needed, according to the scoring instructions provided by HealthMeasures.

#### Symptom Outcomes

General symptoms were assessed using PROMIS measures, including the frequency of anxiety [26], depression [27], and fatigue [28], as well as the severity of pain [29] and sleep disturbance [30]. For each measure, a PROMIS score was calculated using the HealthMeasures Scoring Service administration platform, according to the scoring instructions provided by HealthMeasures.

Prostate cancer-specific symptoms were measured using the 26-item Expanded Prostate Cancer Index Composite (EPIC-26), which evaluates patients’ function and symptom-related bother across urinary, bowel, sexual, and hormonal domains [31]. Patients self-reported their symptoms, while partners completed a 4-item EPIC-Spouse questionnaire [32] to assess the extent to which the patients’ PCa symptoms were bothersome or burdensome to them.

#### Factors Potentially Influencing the Outcomes

Demographic characteristics were collected at baseline, including age, gender, race, patient’s treatment type (surgery or radiation), and other sociodemographic variables, such as marital status and annual income for both patients and partners.

The Charlson Comorbidity Index was administered to patients and partners at baseline. The Charlson Comorbidity Index score indicates the number of comorbid conditions that an individual reports.

### Data Analysis

#### Overview

We summarized participants’ baseline characteristics and outcome variables with percentages for categorical variables and means with SDs for continuous variables. Following the intention-to-treat principle, we analyzed patients and their partners based on the assigned study group rather than their completion of the intervention.

To evaluate the efficacy of PERC vs the control group, a separate multilevel linear mixed model (MLMM) was fit to the longitudinal data for each outcome of interest. Outcomes (eg, QOL and symptom outcomes), assessed at 4, 8, and 12 months post baseline (T2-T4), were modeled as dependent variables, with time treated as a categorical variable. The MLMMs accounted for the hierarchical structure of the data by partitioning variability at three levels: within-individual (repeated measures over time), within-dyad (role: patient vs partner), and between-dyad. Random effects included a random intercept for each dyad to account for correlations between patients and partners within the same dyad and a random intercept for each individual to account for correlations across repeated measures. Fixed effects included study group, time, role, and all relevant 2-way and 3-way interaction terms (group × time, group × role, time × role, and group × time × role). To control for potential confounding, models were further adjusted for baseline characteristics that differed significantly between study groups, as well as baseline outcome scores [25]. Group differences at baseline were evaluated separately for patients and partners using the Pearson chi-square test for categorical variables and the Wilcoxon rank-sum test for continuous variables.

From the MLMMs, we estimated adjusted marginal means and SDs for each outcome. We examined three sets of effects: (1) group effects averaged across time, (2) time effects averaged across study groups, and (3) group effects at each time point, separately for patients and partners. For each set of effects, we summarized the adjusted marginal means and SDs for the relevant groups or time points. Pairwise comparisons were conducted, and for each comparison, we reported the mean difference, 95% CI, *P* value, and effect size (Cohen *d*). Effect sizes were interpreted using conventional benchmarks for Cohen *d* (0.2=small, 0.5=medium, and 0.8=large) [31]. In addition, consistent with commonly used distribution-based approaches for health-related QOL outcomes, an absolute effect size of |*d*|≥0.5 was considered indicative of a potentially clinically meaningful difference, as a change of approximately 0.5 SDs has often been associated with patient-perceived meaningful change [32,33].

For all primary and secondary outcomes, we evaluated the effects of group or time on outcomes of interest, applying a Bonferroni correction to account for testing patients and partners separately, thereby controlling the family-wise type I error rate. A 2-sided *P*<.025 was considered statistically significant. For the FACT-G, we adopted a hierarchical testing approach. Specifically, we first tested the effect of group or time on the FACT-G total score. The 4 FACT-G subdomains and other secondary outcomes were evaluated subsequently, with interpretation depending on the total score test result. If the effect on the FACT-G total score was statistically significant, the subdomain tests were considered formal and confirmatory. If the effect on the FACT-G total score was not significant, the subdomain results were presented descriptively and treated as exploratory rather than formal hypothesis tests. Selected baseline demographic and comorbidity variables were included as covariates in the MLMM models to improve precision and adjust for potential baseline imbalances.

All statistical analyses were performed using R software (version 4.1.2; R Core Team, R Foundation for Statistical Computing). For analyses not applying a Bonferroni correction, a significance level of .05 was used.

#### Missing Data Handling and Sensitivity Analysis

The primary analyses assumed that data were missing at random, which allows valid estimation in the presence of incomplete longitudinal data. To evaluate the robustness of the findings to missingness, sensitivity analyses were conducted using a complete-case approach. The analyses were restricted to dyads with complete follow-up data across all assessment time points, and the same model specifications used in the primary analyses were refitted.

#### Power Analysis and Sample Size Calculation

We calculated power for comparing our primary outcome (overall QOL) between groups using a standard approach for linear mixed models. Because we assessed outcomes for patients and partners separately, we applied a Bonferroni correction to allow for separate overall tests for patients and partners, each with 2-sided α=.025. This approach was used because conclusions may differ for patients and partners, although dyadic data were modeled simultaneously. Based on our pilot study, we assumed a common SD for the overall QOL scores of 15 points and a within-person correlation between repeated measurements of 0.75. We accounted for a 7% loss to follow-up every 4 months, for a total attrition of 20% through 12 months.

Under these assumptions, based on our preliminary studies, we report power for 2 scenarios: first, allowing for attenuating effects and then assuming constant effects. For the first scenario, we assumed that, on average, PERC would result in improved QOL relative to the control condition, but that these benefits might realistically be expected to decrease somewhat over time. Assuming that the mean difference between groups would be 7.5 points in overall QOL scores at 4 months (ie, a moderate effect size of 0.5), this would represent a clinically meaningful difference immediately following the intervention. We expected that this effect would decrease by 15% every 4 months. Randomizing 125 dyads per group would provide 90% power to reject the overall null hypothesis of no differences between groups across all time points. Under this scenario, this sample size would provide 94% power for the 4-month comparison, 83% power for the 8-month comparison, and 51% power for the 12-month comparison. For the second scenario, we assumed that the intervention would have a constant effect of 6.5 points at each time point (an effect size of 0.43, representing a small-to-moderate effect). This sample size provided at least 80% power for each comparison.

### Ethical Considerations

The study was approved by the Institutional Review Board at the University of North Carolina at Chapel Hill (IRB 17‐0482) and the Institutional Review Board at the University of Texas Health Science Center at San Antonio (IRB 23‐0191).

## Results

### Overview

**Figure 1.**
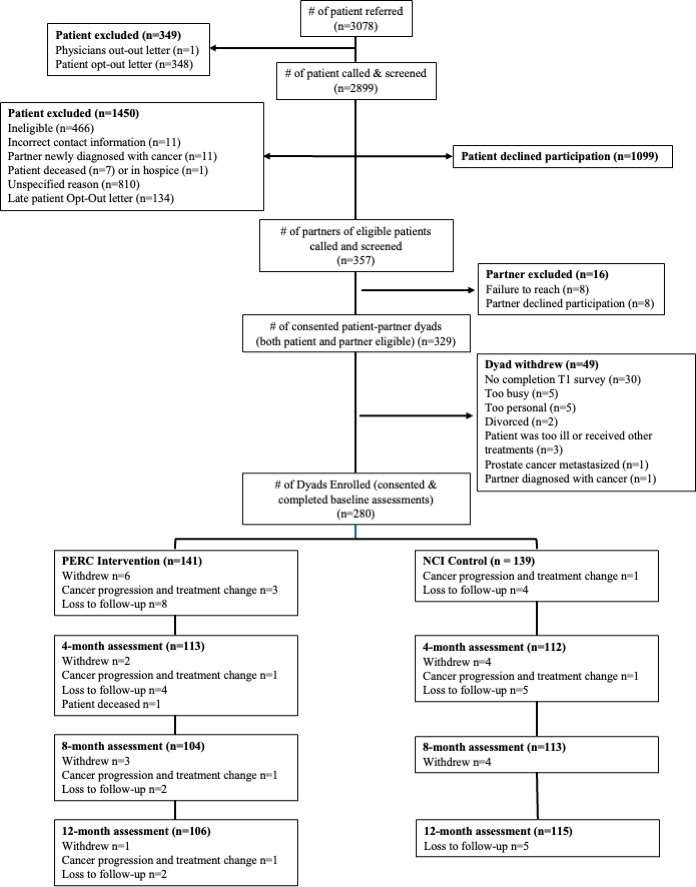
CONSORT (Consolidated Standards of Reporting Trials) flow diagram. NCI: National Cancer Institute; PERC: Prostate Cancer Education and Resources for Couples. Note: Any apparent discrepancies in the number of contacts are attributable to the dynamic nature of participant enrollment and follow-up.

Between 2018 and 2021, coinciding with the onset of COVID-19, 280 eligible, consented dyads completed baseline surveys (enrollment rate: 280/329, 85.1%) and were randomly assigned to either the PERC (n=141) or control group (n=139) on a 1:1 basis [33]. Demographic data are shown in Multimedia Appendix 1. A total of 221 (78.9%) dyads completed the 12-month follow-up (T4) survey (PERC=106 and control=115; Figure 1). Descriptive results for outcomes of interest are detailed in Multimedia Appendix 2. Estimation results for the fixed effects from the MLMM for the FACT-G total score are provided in Multimedia Appendix 3.

Among baseline characteristics, income was the only variable that differed significantly between patients in the PERC and NCI control groups (*χ*²_2_=6.34; *P*=.04). To account for this baseline imbalance and ensure valid group comparisons, income category was included as a covariate in all MLMM analyses.

### QOL Outcomes

As shown in Tables 1-3, no statistically significant differences were observed in FACT-G total scores between groups (primary outcomes), either averaged across time or at individual time points, for patients or partners. Similarly, no significant group differences were detected in any of the 4 FACT-G subdomains (secondary outcomes). At T4, patients in the PERC group reported slightly higher physical well-being scores compared with the control group (mean difference 0.9, 95% CI –0.1 to 1.9; *P*=.04), although the CI included zero. The effect size was *d*=0.33, indicating a small-to-moderate effect size. Although this did not reach the |*d*|≥0.5 threshold for clinical meaningfulness, it suggests a positive trend in the expected direction.

**Table 1.** Patients’ and partners’ outcomes: group effects by role averaged across time (based on multilevel linear mixed models).

Outcome	Patients					Partners				
	PERC^a^, mean (SD)	Control, mean (SD)	Difference (95% CI^b^)	*P* value^c^	Effect size^d^	PERC, mean (SD)	Control, mean (SD)	Difference (95% CI^b^)	*P* value^c^	Effect size^d^
Primary outcomes										
QOL^e^ FACT-G^f^										
FACT-G total score	90.6 (13.9)	89.4 (14.4)	1.2 (−1.2 to 3.6)	.27	0.10	87.8 (13.8)	87.7 (13.6)	0.1 (−2.2 to 2.5)	.89	0.01
Secondary outcomes										
QOL outcomes subdomains^g^										
Physical	25.0 (4.5)	24.5 (4.6)	0.5 (−0.3 to 1.3)	.15	0.12	23.9 (4.4)	23.8 (4.3)	0.1 (−0.6 to 0.9)	.73	0.03
Social	22.3 (5.4)	21.9 (5.6)	0.4 (−0.5 to 1.3)	.33	0.08	22.4 (5.4)	22.0 (5.3)	0.4 (−0.5 to 1.3)	.31	0.09
Emotional	20.8 (4.3)	20.6 (4.4)	0.2 (−0.5 to 0.9)	.58	0.05	20.1 (4.2)	20.2 (4.2)	−0.1 (−0.8 to 0.6)	.80	−0.02
Functional	22.4 (5.5)	22.2 (5.7)	0.2 (−0.7 to 1.2)	.60	0.04	21.3 (5.5)	21.4 (5.4)	−0.1 (−1.0 to 0.9)	.85	−0.02
Psychosocial outcomes										
Appraisal of illness^g^	3.9 (0.8)	3.8 (0.8)	0.1 (−0.0 to 0.2)	.08	0.15	3.9 (0.8)	3.9 (0.8)	0.0 (−0.1 to 0.1)	.97	0.00
Coping resources^g^										
Cancer Self-Efficacy Scale	78.3 (15.4)	78.0 (15.9)	0.3 (−2.3 to 2.9)	.78	0.02	79.2 (15.3)	77.9 (14.9)	1.3 (−1.3 to 3.9)	.28	0.09
Social support^g^										
Emotional support	55.8 (9.1)	54.9 (9.4)	0.9 (−0.6 to 2.5)	.18	0.11	53.6 (9.0)	54.2 (8.8)	−0.6 (−2.1 to 0.9)	.37	−0.08
Informational support	56.1 (10.3)	56.3 (10.7)	−0.1 (−1.9 to 1.6)	.88	−0.01	56.4 (10.2)	55.6 (10.0)	0.8 (−1.0 to 2.5)	.32	0.09
Instrumental support	58.9 (8.4)	58.3 (8.7)	0.6 (−0.8 to 2.1)	.31	0.09	56.6 (8.3)	56.5 (8.2)	0.1 (−1.3 to 1.5)	.84	0.02
System outcomes (general symptoms^h^)										
Anxiety	46.4 (11.1)	46.7 (11.4)	−0.3 (−2.2 to 1.5)	.69	−0.03	47.6 (10.9)	47.6 (10.8)	−0.1 (−1.9 to 1.8)	.95	−0.01
Depression	45.5 (10.3)	45.9 (10.7)	−0.4 (−2.2 to 1.3)	.58	−0.05	47.0 (10.2)	46.1 (10.0)	1.0 (−0.8 to 2.7)	.21	0.11
Pain	47.4 (11.1)	48.8 (11.4)	−1.3 (−3.2 to 0.5)	.11	−0.14	51.0 (10.9)	51.3 (10.8)	−0.2 (−2.1 to 1.6)	.78	−0.02
Sleep	47.4 (11.5)	48.6 (11.9)	−1.2 (−3.1 to 0.8)	.18	−0.12	50.1 (11.3)	50.3 (11.1)	−0.1 (−2.1 to 1.8)	.87	−0.01
Fatigue	45.7 (10.3)	47.8 (10.7)	−2.1 (−3.9 to −0.4)	.007	−0.23	48.2 (10.2)	48.1 (10.1)	0.0 (−1.7 to 1.8)	.96	0.00
PCa^i^-specific symptoms: EPIC^j^										
Urinary	83.0 (26.5)	81.4 (27.6)	1.6 (−3.0 to 6.3)	.43	0.07	85.8 (25.4)	82.2 (25.0)	3.6 (−1.2 to 8.5)	.09	0.16
Bowel	93.8 (18.0)	92.6 (18.6)	1.2 (−1.8 to 4.3)	.37	0.08	95.7 (17.5)	92.9 (17.0)	2.7 (−0.6 to 6.1)	.06	0.18
Sexual	39.8 (42.9)	43.4 (43.9)	−3.7 (−10.6 to 3.3)	.24	−0.10	53.2 (39.7)	50.6 (39.5)	2.6 (−4.7 to 9.9)	.43	0.08
Hormonal	84.5 (31.0)	84.3 (32.0)	0.2 (−5.0 to 5.4)	.93	0.01	79.3 (30.0)	76.7 (29.2)	2.6 (−3.1 to 8.2)	.31	0.10

a PERC: Prostate Cancer Education and Resources for Couples.

b The 95% CIs represent Bonferroni-corrected simultaneous CIs for the mean differences between two groups, reported separately for patients and partners. CIs that do not include zero indicate statistically significant differences between groups.

c The *P *values correspond to 2-sided tests of the null hypothesis that the mean difference between two groups equals zero. After applying Bonferroni correction for tests conducted separately in patients and partners, a *P* value less than 0.025 is considered statistically significant.

d Effect sizes (Cohen *d*) are interpreted as small (0.2), medium (0.5), and large (0.8). Effects with |*d*|≥0.5 are considered potentially clinically meaningful.

e QOL: quality of life.

f FACT-G: Functional Assessment of Chronic Illness Therapy-General.

g Higher scores indicated more positive outcomes: ie, better quality of life, better perception of threat of symptoms, less severe symptoms, greater self-efficacy in symptom management, more social support, and better interpersonal support.

h Higher scores indicated more negative outcomes: ie, more frequent or severe symptoms.

i PCa: prostate cancer.

j The EPIC-26 (26-item Expanded Prostate Cancer Index Composite) scores for patients and partners were standardized to enable direct comparison in subsequent analyses.

**Table 2.** Patients’ and partners’ outcomes: group effects by time and role at T2 and T3 (based on multilevel linear mixed models).

Outcome	Patients					Partners				
	PERC^a^, mean (SD)	Control, mean (SD)	Difference (95% CI^b^)	*P* value^c^	Effect size^d^	PERC, mean (SD)	Control, mean (SD)	Difference (95% CI^b^)	*P* value^c^	Effect size^d^
Time: T2										
Primary outcomes: QOL^e^ FACT-G^f^										
FACT-G total score	90.0 (9.8)	90.6 (10.1)	−0.6 (−3.5 to 2.3)	.64	−0.07	89.3 (9.7)	89.4 (9.5)	−0.1 (−2.9 to 2.7)	.94	−0.01
Secondary outcomes: QOL subdomains^g^										
Physical	24.6 (3.2)	24.5 (3.2)	0.0 (−0.9 to 1.0)	.92	0.01	24.0 (3.1)	23.8 (3.1)	0.1 (−0.8 to 1.0)	.77	0.04
Social	22.5 (3.9)	22.3 (4.0)	0.2 (−1.0 to 1.3)	.73	0.05	23.0 (3.8)	22.4 (3.8)	0.6 (−0.5 to 1.7)	.25	0.16
Emotional	20.7 (3.1)	20.6 (3.2)	0.1 (−0.8 to 1.0)	.80	0.04	20.3 (3.1)	20.3 (3.0)	0.0 (−0.9 to 0.9)	.93	0.01
Functional	22.0 (3.9)	22.7 (4.0)	−0.7 (−1.9 to 0.4)	.16	−0.20	22.0 (3.9)	22.5 (3.8)	−0.6 (−1.7 to 0.6)	.25	−0.16
Secondary outcomes: psychosocial outcomes										
Appraisals^g^										
Appraisal of Illness	3.9 (0.6)	3.8 (0.6)	0.1 (−0.1 to 0.3)	.19	0.19	3.9 (0.6)	3.9 (0.5)	0.0 (−0.2 to 0.2)	.99	0.00
Coping resources^g^										
Cancer Self-Efficacy Scale	78.9 (11.2)	79.0 (11.4)	−0.1 (−3.4 to 3.1)	.94	−0.01	80.6 (11.2)	77.7 (10.9)	2.9 (−0.4 to 6.2)	.05	0.28
Social support^g^										
Emotional support	55.3 (6.6)	55.0 (6.7)	0.3 (−1.6 to 2.2)	.70	0.05	54.1 (6.5)	54.8 (6.4)	−0.7 (−2.6 to 1.2)	.40	−0.12
Informational support	56.0 (7.6)	56.1 (7.8)	−0.1 (−2.4 to 2.1)	.89	−0.02	56.3 (7.6)	55.6 (7.4)	0.7 (−1.6 to 2.9)	.50	0.10
Instrumental support	58.9 (6.2)	58.4 (6.3)	0.4 (−1.3 to 2.2)	.58	0.08	58.0 (6.1)	56.5 (6.0)	1.5 (−0.3 to 3.3)	.06	0.26
Secondary outcomes: symptom outcomes										
General symptoms^h^										
Anxiety	46.8 (7.9)	46.4 (8.0)	0.4 (−1.9 to 2.7)	.71	0.05	47.4 (7.8)	47.0 (7.7)	0.5 (−1.8 to 2.7)	.65	0.06
Depression	45.7 (7.2)	45.5 (7.3)	0.2 (−1.8 to 2.3)	.81	0.03	46.4 (7.1)	45.5 (7.0)	0.9 (−1.1 to 3.0)	.32	0.14
Pain	48.1 (8.0)	47.5 (8.2)	0.6 (−1.7 to 2.9)	.58	0.08	50.0 (7.9)	50.2 (7.8)	−0.2 (−2.5 to 2.1)	.86	−0.02
Sleep	48.0 (8.1)	48.6 (8.2)	−0.6 (−2.9 to 1.7)	.57	−0.08	50.2 (8.0)	50.9 (7.8)	−0.7 (−3.1 to 1.6)	.47	−0.10
Fatigue	45.6 (7.5)	47.0 (7.7)	−1.4 (−3.6 to 0.8)	.15	−0.20	47.6 (7.4)	47.2 (7.3)	0.5 (−1.7 to 2.6)	.64	0.07
PCa^i^-specific symptoms: EPIC^j^										
Urinary	81.3 (18.6)	80.0 (18.9)	1.3 (−4.2 to 6.9)	.59	0.08	84.3 (18.5)	84.9 (18.2)	−0.7 (−6.0 to 4.7)	.78	−0.04
Bowel	93.1 (13.5)	92.4 (13.8)	0.7 (−3.2 to 4.7)	.69	0.06	96.6 (13.3)	95.2 (13.1)	1.4 (−2.6 to 5.3)	.44	0.11
Sexual	37.4 (29.3)	41.8 (29.7)	−4.4 (−12.6 to 3.8)	.23	−0.18	53.6 (28.6)	50.3 (28.2)	3.3 (−4.8 to 11.3)	.36	0.13
Hormonal	82.0 (22.6)	85.0 (23.0)	−3.0 (−9.6 to 3.5)	.30	−0.15	79.9 (22.3)	75.2 (21.9)	4.7 (−1.8 to 11.3)	.11	0.23
Time: T3										
Primary outcomes QOL FACT-G^g^										
FACT-G total score	91.1 (9.6)	89.3 (9.9)	1.9 (−1.1 to 4.8)	.17	0.21	87.8 (9.5)	87.8 (9.5)	0.0 (−3.0 to 3.0)	.98	0.00
Secondary outcomes: QOL subdomains^g^										
Physical	25.0 (3.1)	24.5 (3.2)	0.5 (−0.4 to 1.5)	.23	0.18	23.8 (3.0)	23.9 (3.0)	−0.1 (−1.1 to 0.9)	.81	−0.04
Social	22.5 (3.8)	21.8 (3.9)	0.7 (−0.5 to 1.9)	.18	0.20	22.5 (3.8)	22.0 (3.8)	0.5 (−0.7 to 1.7)	.34	0.14
Emotional	20.8 (3.0)	20.7 (3.1)	0.1 (−0.9 to 1.0)	.82	0.03	20.1 (3.0)	20.4 (3.0)	−0.3 (−1.2 to 0.7)	.48	−0.11
Functional	22.6 (3.8)	22.0 (4.0)	0.6 (−0.6 to 1.8)	.27	0.17	21.4 (3.8)	21.2 (3.8)	0.1 (−1.1 to 1.3)	.80	0.04
Secondary outcomes: psychosocial outcomes										
Appraisals^g^										
Appraisal of illness	3.8 (0.5)	3.8 (0.6)	0.0 (−0.1 to 0.2)	.74	0.05	3.9 (0.5)	3.9 (0.5)	−0.0 (−0.2 to 0.1)	.65	−0.07
Coping resources^g^										
Cancer Self-Efficacy Scale	78.8 (11.0)	77.8 (11.3)	1.0 (−2.4 to 4.4)	.52	0.10	77.8 (11.0)	78.9 (10.8)	−1.1 (−4.5 to 2.3)	.48	−0.11
Social support^g^										
Emotional support	56.4 (6.4)	54.9 (6.6)	1.4 (−0.6 to 3.5)	.11	0.24	53.4 (6.4)	54.6 (6.4)	−1.2 (−3.2 to 0.8)	.17	−0.21
Informational support	56.7 (7.5)	56.4 (7.7)	0.3 (−2.1 to 2.6)	.80	0.04	57.1 (7.5)	56.1 (7.4)	1.0 (−1.4 to 3.3)	.36	0.14
Instrumental support	58.6 (6.1)	58.8 (6.2)	−0.2 (−2.1 to 1.7)	.78	−0.04	56.2 (6.0)	56.7 (6.0)	−0.5 (−2.4 to 1.3)	.52	−0.10
Secondary outcomes: symptom outcomes										
General symptoms^h^										
Anxiety	46.3 (7.7)	46.5 (7.9)	−0.2 (−2.6 to 2.2)	.86	−0.03	48.1 (7.6)	47.2 (7.7)	0.9 (−1.5 to 3.3)	.40	0.13
Depression	45.9 (7.0)	45.8 (7.2)	0.2 (−2.0 to 2.3)	.86	0.03	47.0 (6.9)	45.2 (6.9)	1.8 (−0.3 to 4.0)	.06	0.29
Pain	47.7 (7.9)	49.6 (8.1)	−1.9 (−4.3 to 0.6)	.09	−0.25	51.4 (7.8)	51.1 (7.8)	0.3 (−2.2 to 2.7)	.79	0.04
Sleep	47.3 (7.9)	48.4 (8.1)	−1.1 (−3.6 to 1.3)	.30	−0.16	50.7 (7.8)	49.9 (7.8)	0.8 (−1.6 to 3.3)	.44	0.12
Fatigue	46.4 (7.4)	47.6 (7.6)	−1.2 (−3.6 to 1.1)	.23	−0.18	48.5 (7.3)	48.6 (7.3)	−0.0 (−2.4 to 2.3)	.97	−0.01
PCa-specific symptoms: EPIC^g^										
Urinary	84.8 (18.0)	82.6 (18.8)	2.2 (−3.5 to 8.0)	.38	0.13	88.1 (15.4)	81.5 (16.0)	6.5 (−1.0 to 14.1)	.05	0.44
Bowel	93.8 (13.3)	93.2 (13.7)	0.7 (−3.5 to 4.8)	.72	0.05	96.6 (12.1)	92.2 (12.3)	4.4 (−1.5 to 10.4)	.10	0.37
Sexual	41.0 (28.4)	41.8 (29.0)	−0.8 (-9.4 to 7.8)	.83	−0.03	55.3 (23.2)	50.8 (24.4)	4.6 (−7.0 to 16.2)	.38	0.21
Hormonal	84.9 (22.1)	84.4 (22.8)	0.4 (−6.5 to 7.4)	.89	0.02	80.2 (19.8)	74.0 (20.2)	6.2 (−3.5 to 15.8)	.15	0.32

a PERC: Prostate Cancer Education and Resources for Couples.

b The 95% CIs represent Bonferroni-corrected simultaneous CIs for the mean differences between 2 groups, reported separately for patients and partners. CIs that do not include 0 indicate statistically significant differences between groups.

c The *P* values correspond to 2-sided tests of the null hypothesis that the mean difference between 2 groups equals 0. After applying Bonferroni correction for tests conducted separately in patients and partners, a *P* value less than .025 is considered statistically significant.

d Effect sizes (Cohen *d*) are interpreted as small (0.2), medium (0.5), and large (0.8). Effects with |*d*|≥0.5 are considered potentially clinically meaningful.

e QOL: quality of life

f FACT-G: Functional Assessment of Chronic Illness Therapy-General.

g Higher scores indicated more positive outcomes, ie, better quality of life, better perception of threat of symptoms, less severe symptoms, greater self-efficacy in symptom management, more social support, and better interpersonal support.

h Higher scores indicated more negative outcomes: ie, more frequent or severe symptoms.

i PCa: prostate cancer.

j The EPIC-26 (26-item Expanded Prostate Cancer Index Composite) scores for patients and partners were standardized to enable direct comparison in subsequent analyses.

**Table 3.** Patients’ and partners’ outcomes: group effects by time and role at T4 (based on multilevel linear mixed models).

Outcome	Patients					Partners				
	PERC^a^, mean (SD)	Control, mean (SD)	Difference (95% CI^b^)	*P* value^c^	Effect size^d^	PERC, mean (SD)	Control, mean (SD)	Difference (95% CI^b^)	*P* value^c^	Effect size^d^
Time: T4										
Primary outcomes QOL^e^ FACT-G^f^										
FACT-G total score	90.7 (9.5)	88.4 (9.7)	2.3 (−0.8 to 5.3)	.10	0.26	86.2 (9.4)	85.8 (9.4)	0.5 (−2.6, 3.5)	.72	0.06
Secondary outcomes: QOL subdomains^g^										
Physical	25.3 (3.1)	24.4 (3.1)	0.9 (−0.1 to 1.9)	.04	0.33	23.9 (3.0)	23.6 (3.0)	0.3 (−0.7 to 1.3)	.45	0.12
Social	21.8 (3.8)	21.5 (3.8)	0.3 (−0.9 to 1.5)	.57	0.09	21.7 (3.7)	21.6 (3.7)	0.2 (−1.1 to 1.4)	.77	0.04
Emotional	20.9 (3.0)	20.5 (3.1)	0.3 (−0.6 to 1.3)	.42	0.12	19.9 (3.0)	19.8 (3.0)	0.0 (−0.9 to 1.0)	.96	0.01
Functional	22.6 (3.8)	21.8 (3.9)	0.8 (−0.5 to 2.0)	.16	0.22	20.7 (3.8)	20.5 (3.8)	0.2 (−1.0 to 1.4)	.69	0.06
Secondary outcomes: psychosocial outcomes										
Appraisals^g^										
Appraisal of illness	3.9 (0.5)	3.7 (0.6)	0.2 (0.0 to 0.4)	.02	0.38	3.8 (0.5)	3.8 (0.5)	0.0 (−0.1 to 0.2)	.61	0.08
Coping resources^g^										
Cancer Self-Efficacy Scale	77.3 (10.9)	77.2 (11.1)	0.1 (−3.4 to 3.6)	.94	0.01	79.1 (10.9)	77.1 (10.8)	2.0 (−1.5 to 5.5)	.21	0.20
Social support^g^										
Emotional support	55.7 (6.4)	54.7 (6.5)	1.0 (−1.1 to 3.0)	.29	0.17	53.2 (6.3)	53.0 (6.3)	0.1 (−1.9 to 2.2)	.91	0.02
Informational support	55.7 (7.5)	56.2 (7.6)	−0.5 (−2.9 to 1.9)	.65	−0.07	55.8 (7.4)	55.1 (7.4)	0.7 (−1.7 to 3.1)	.52	0.10
Instrumental support	59.3 (6.0)	57.5 (6.1)	1.7 (−0.2 to 3.7)	.05	0.31	55.7 (6.0)	56.2 (5.9)	−0.5 (−2.5 to 1.4)	.53	−0.10
Secondary outcomes: symptom outcomes										
General symptoms^h^										
Anxiety	46.1 (7.7)	47.3 (7.8)	−1.2 (−3.7 to 1.3)	.28	−0.17	47.3 (7.6)	48.8 (7.6)	−1.5 (−4.0 to 0.9)	.16	−0.22
Depression	44.8 (6.9)	46.5 (7.1)	−1.7 (−3.9 to 0.5)	.09	−0.26	47.6 (6.8)	47.5 (6.8)	0.1 (−2.1 to 2.3)	.90	0.02
Pain	46.6 (7.8)	49.3 (8.0)	−2.7 (−5.3 to −0.2)	.02	−0.38	51.7 (7.7)	52.5 (7.7)	−0.8 (−3.3 to 1.7)	.47	−0.11
Sleep	47.0 (7.8)	48.7 (8.0)	−1.8 (−4.3 to 0.7)	.11	−0.25	49.5 (7.7)	50.0 (7.7)	−0.5 (−3.0 to 2.0)	.64	−0.07
Fatigue	45.1 (7.3)	48.8 (7.5)	−3.7 (−6.1 to −1.4)	<.001	−0.55	48.4 (7.2)	48.7 (7.3)	−0.3 (−2.6 to 2.1)	.79	−0.04
PCa^i^-specific symptoms: EPIC^j^										
Urinary	83.0 (17.6)	81.6 (18.3)	1.3 (−4.6 to 7.3)	.62	0.08	85.2 (17.8)	80.2 (17.7)	5.0 (−0.7 to 10.7)	.05	0.30
Bowel	94.6 (13.2)	92.4 (13.5)	2.3 (−2.0 to 6.6)	.23	0.19	93.9 (13.1)	91.4 (13.1)	2.5 (−1.8 to 6.7)	.20	0.20
Sexual	40.8 (27.8)	46.6 (28.6)	−5.7 (−14.6 to 3.1)	.14	−0.24	50.6 (27.6)	50.8 (27.7)	−0.2 (−8.8 to 8.4)	.96	−0.01
Hormonal	86.6 (21.9)	83.5 (22.4)	3.2 (−3.9 to 10.3)	.32	0.16	77.7 (21.9)	80.9 (21.7)	−3.2 (−10.2 to 3.9)	.31	−0.16

a PERC: Prostate Cancer Education and Resources for Couples.

b The 95% CIs represent Bonferroni-corrected simultaneous CIs for the mean differences between 2 groups, reported separately for patients and partners. CIs that do not include 0 indicate statistically significant differences between groups.

c The *P* values correspond to 2-sided tests of the null hypothesis that the mean difference between 2 groups equals 0. After applying Bonferroni correction for tests conducted separately in patients and partners, a *P* value less than .025 is considered statistically significant.

d Effect sizes (Cohen *d*) are interpreted as small (0.2), medium (0.5), and large (0.8). Effects with |*d*|≥0.5 are considered potentially clinically meaningful.

e QOL: quality of life.

f FACT-G: Functional Assessment of Chronic Illness Therapy-General.

g Higher scores indicated more positive outcomes, ie, better quality of life, better perception of threat of symptoms, less severe symptoms, greater self-efficacy in symptom management, more social support, and better interpersonal support.

h Higher scores indicated more negative outcomes, ie, more frequent or severe symptoms.

i PCa: prostate cancer.

j The EPIC-26 (26-item Expanded Prostate Cancer Index Composite) scores for patients and partners were standardized to enable direct comparison in subsequent analyses.

### Psychosocial Outcomes

As shown in Table 1, no significant group differences were observed for psychosocial outcomes when averaged across time. However, at T4, patients in the PERC group reported significantly higher appraisal of illness (ie, less negative perceptions of PCa) compared with the control group (mean difference 0.2, 95% CI 0.0-0.4, *P*=.02). This corresponds to an effect size of *d*=0.38, indicating a small-to-moderate effect size. Although this did not reach the |*d*|≥0.5 threshold for clinical meaningfulness, it represents a statistically significant improvement in the expected direction.

### General Symptoms

Patients in the PERC group reported significantly less frequent fatigue compared with the control group when averaged across time (mean difference –2.1, 95% CI –3.9 to –0.4; *P*=.007). This difference was most notable at T4, with patients in the PERC group reporting less frequent fatigue than those in the control group (mean difference –3.7, 95% CI –6.1 to –1.4; *P*<.001), corresponding to an effect size of *d*=–0.55. This represents a moderate effect size and exceeds the |*d*|≥0.5 threshold for a clinically meaningful difference. Although no significant group differences in the severity of pain were observed when averaged across time, patients in the PERC group reported significantly less severe pain than the control group at T4 (mean difference –2.7, 95% CI –5.3 to –0.2; *P*=.01). This corresponds to an effect size of *d*=–0.38, indicating a small-to-moderate effect size. Although this did not reach the threshold for clinical meaningfulness (|*d*|≥0.5), it suggests a statistically significant improvement in the expected direction.

### PCa-Specific Symptoms

No significant group differences were observed for any PCa-specific symptoms, either when averaged across time or at individual time points, for patients or partners. However, exploratory differences between groups were observed at certain time points (Table 2). For example, partners in the PERC group reported less bother about patients’ urinary symptoms at T3 compared with those in the control group (mean difference 6.5, 95% CI –1.0 to 14.1; *P*=.05), corresponding to an effect size of *d*=0.44, indicating a small-to-moderate effect size. Although this did not reach the threshold for clinical meaningfulness (|*d*|≥0.5), the magnitude of the effect approaches this threshold and suggests a favorable trend.

### Time Effects

Based on the fixed-effects estimates from the MLMM for the FACT-G total score (Multimedia Appendix 3), the FACT-G total score at T4 was significantly lower than at T2 (estimate=–3.67; *P*<.001), controlling for other variables. As shown in Tables 4-6, time effects on the FACT-G total score were significant for partners across all pairwise comparisons (*P*=.02 for T3 vs T2, *P*<.001 for T4 vs T2, and *P*=.01 for T4 vs T3).

**Table 4.** Patients’ and partners’ outcomes: time effects by role averaged across study groups for T3 vs T2 (based on multilevel linear mixed models).

Outcome	Patients					Partners				
	T3, mean (SD)	T2, mean (SD)	Difference (95% CI^a^)	*P* value^b^	Effect size^c^	T3, mean (SD)	T2, mean (SD)	Difference (95% CI^a^)	*P* value^b^	Effect size^c^
T3 vs T2										
Primary outcomes: QOL^d^ FACT-G^e^										
FACT-G total score	90.2 (10.6)	90.3 (10.8)	−0.1 (−1.6 to 1.5)	.94	−0.01	87.8 (10.2)	89.4 (10.3)	−1.6 (−3.1 to −0.1)	.02	−0.24
Secondary outcomes: QOL subdomains^f^										
Physical	24.8 (3.4)	24.5 (3.5)	0.2 (−0.3 to 0.7)	.28	0.11	23.8 (3.3)	23.9 (3.3)	−0.1 (−0.6 to 0.4)	.66	−0.04
Social	22.2 (4.2)	22.4 (4.3)	−0.2 (−0.9 to 0.4)	.40	−0.09	22.2 (4.0)	22.7 (4.1)	−0.5 (−1.1 to 0.1)	.06	−0.19
Emotional	20.7 (3.3)	20.7 (3.4)	0.0 (−0.5 to 0.6)	.84	0.02	20.3 (3.2)	20.3 (3.2)	−0.1 (−0.6 to 0.5)	.81	−0.02
Functional	22.3 (4.2)	22.4 (4.3)	−0.0 (−0.7 to 0.6)	.88	−0.02	21.3 (4.1)	22.3 (4.1)	−1.0 (−1.6 to −0.3)	<.001	−0.36
Secondary outcomes: psychosocial outcomes										
Appraisals^f^										
Appraisal of illness	3.8 (0.6)	3.9 (0.6)	−0.0 (−0.1 to 0.1)	.32	−0.10	3.9 (0.6)	3.9 (0.6)	−0.1 (−0.2 to 0.0)	.13	−0.16
Coping resources^f^										
Cancer Self-Efficacy Scale	78.3 (12.1)	78.9 (12.3)	−0.7 (−2.5 to 1.2)	.43	−0.08	78.3 (11.6)	79.1 (11.7)	−0.8 (−2.7 to 1.1)	.34	−0.10
Social support^f^										
Emotional support	55.7 (7.1)	55.2 (7.2)	0.5 (−0.6 to 1.6)	.29	0.11	54.0 (6.8)	54.5 (6.9)	−0.5 (−1.6 to 0.6)	.30	−0.11
Informational support	56.6 (8.2)	56.1 (8.3)	0.5 (−0.8 to 1.8)	.40	0.09	56.6 (7.9)	55.9 (8.0)	0.7 (−0.6 to 2.0)	.24	0.12
Instrumental support	58.7 (6.7)	58.7 (6.8)	0.0 (−1.0 to 1.1)	.92	0.01	56.5 (6.4)	57.3 (6.5)	−0.8 (−1.8 to 0.3)	.09	−0.17
Secondary outcomes: symptom outcomes										
General symptoms^g^										
Anxiety	46.4 (8.5)	46.6 (8.7)	−0.2 (−1.4 to 1.1)	.76	−0.03	47.6 (8.2)	47.2 (8.3)	0.4 (−0.8 to 1.7)	.43	0.08
Depression	45.9 (7.7)	45.6 (7.9)	0.3 (−0.8 to 1.4)	.56	0.06	46.1 (7.4)	46.0 (7.5)	0.1 (−0.9 to 1.2)	.77	0.03
Pain	48.6 (8.6)	47.8 (8.8)	0.8 (−0.5 to 2.2)	.15	0.15	51.2 (8.3)	50.1 (8.4)	1.1 (−0.2 to 2.4)	.06	0.19
Sleep	47.8 (8.7)	48.3 (8.9)	−0.5 (−1.7 to 0.7)	.36	−0.09	50.3 (8.3)	50.5 (8.4)	−0.3 (−1.5 to 1.0)	.65	−0.05
Fatigue	47.0 (8.1)	46.3 (8.2)	0.7 (−0.6 to 1.9)	.24	0.12	48.5 (7.8)	47.4 (7.8)	1.2 (−0.1 to 2.4)	.04	0.21
PCa^h^-specific symptoms: EPIC^i^										
Urinary	83.7 (19.9)	80.7 (20.4)	3.0 (0.2 to 5.9)	.02	0.26	84.8 (16.6)	84.6 (19.6)	0.2 (−3.5 to 4.0)	.89	0.02
Bowel	93.5 (14.5)	92.7 (14.7)	0.8 (−1.6 to 3.2)	.46	0.08	94.4 (12.8)	95.9 (14.0)	−1.5 (−4.7 to 1.7)	.30	−0.14
Sexual	41.4 (32.2)	39.6 (33.3)	1.8 (−2.3 to 6.0)	.32	0.10	53.0 (25.6)	52.0 (31.4)	1.1 (−4.7 to 6.9)	.67	0.06
Hormonal	84.7 (24.2)	83.5 (24.8)	1.2 (−2.6 to 4.9)	.48	0.07	77.1 (21.0)	77.6 (23.6)	−0.5 (−5.6 to 4.6)	.84	−0.03

a The 95% CIs represent Bonferroni-corrected simultaneous CIs for the mean differences between 2 groups, reported separately for patients and partners. CIs that do not include 0 indicate statistically significant differences between groups.

b The *P *values correspond to 2-sided tests of the null hypothesis that the mean difference between 2 groups equals 0. After applying Bonferroni correction for tests conducted separately in patients and partners, a *P* value less than .025 is considered statistically significant.

c Effect sizes (Cohen *d*) are interpreted as small (0.2), medium (0.5), and large (0.8). Effects with |*d*|≥0.5 are considered potentially clinically meaningful.

d QOL: quality of life.

e FACT-G: Functional Assessment of Chronic Illness Therapy-General.

f Higher scores indicated more positive outcomes: ie, better quality of life, better perception of threat of symptoms, less severe symptoms, greater self-efficacy in symptom management, more social support, and better interpersonal support.

g Higher scores indicated more negative outcomes, ie, more frequent or severe symptoms.

h PCa: prostate cancer.

i The EPIC-26 (26-item Expanded Prostate Cancer Index Composite) scores for patients and partners were standardized to enable direct comparison in subsequent analyses.

**Table 5.** Patients’ and partners’ outcomes: time effects by role averaged across study groups for T4 vs T2 (based on multilevel linear mixed models).

Outcome	Patients					Partners				
	T4, mean (SD)	T2, mean (SD)	Difference (95% CI)^a^	*P* value^b^	Effect size^c^	T4, mean (SD)	T2, mean (SD)	Difference (95% CI)	*P* value	Effect size
T4 vs T2										
Primary outcomes: QOL^d^ FACT-G^e^										
FACT-G total score	89.5 (10.4)	90.3 (10.8)	−0.7 (−2.3 to 0.8)	.30	−0.11	86.0 (10.0)	89.4 (10.3)	−3.4 (−4.9 to −1.8)	<.001	−0.51
Secondary outcomes: QOL subdomains										
Physical	24.9 (3.3)	24.5 (3.5)	0.3 (−0.2 to 0.8)	.16	0.15	23.8 (3.2)	23.9 (3.3)	−0.2 (−0.7 to 0.4)	.50	−0.07
Social	21.7 (4.1)	22.4 (4.3)	−0.7 (−1.4 to −0.1)	.009	−0.28	21.7 (4.0)	22.7 (4.1)	−1.1 (−1.7 to −0.4)	<.001	−0.39
Emotional	20.7 (3.3)	20.7 (3.4)	0.0 (−0.5 to 0.5)	.99	0.00	19.9 (3.2)	20.3 (3.2)	−0.5 (−1.0 to 0.0)	.04	−0.22
Functional	22.2 (4.2)	22.4 (4.3)	−0.2 (−0.9 to 0.4)	.44	−0.08	20.6 (4.0)	22.3 (4.1)	−1.7 (−2.3 to −1.0)	<.001	−0.62
Secondary outcomes: psychosocial outcomes										
Appraisals^f^										
Appraisal of illness	3.8 (0.6)	3.9 (0.6)	−0.0 (−0.1 to 0.1)	.49	−0.07	3.8 (0.6)	3.9 (0.6)	−0.1 (−0.2 to −0.1)	<.001	−0.36
Coping resources^f^										
Cancer Self-Efficacy Scale	77.3 (11.9)	78.9 (12.3)	−1.7 (−3.6 to 0.3)	.05	−0.20	78.1 (11.5)	79.1 (11.7)	−1.1 (−3.0 to 0.9)	.21	−0.13
Social support^f^										
Emotional support	55.2 (7.0)	55.2 (7.2)	0.1 (−1.0 to 1.2)	.91	0.01	53.1 (6.7)	54.5 (6.9)	−1.4 (−2.5 to −0.3)	.005	−0.30
Informational support	56.0 (8.1)	56.1 (8.3)	−0.1 (−1.4 to 1.3)	.88	−0.02	55.5 (7.9)	55.9 (8.0)	−0.5 (−1.8 to 0.9)	.44	−0.08
Instrumental support	58.4 (6.6)	58.7 (6.8)	−0.3 (−1.3 to 0.8)	.59	−0.06	55.9 (6.4)	57.3 (6.5)	−1.3 (−2.4 to −0.2)	.006	−0.29
Secondary outcomes: symptom outcomes										
General symptoms^g^										
Anxiety	46.7 (8.3)	46.6 (8.7)	0.1 (−1.2 to 1.4)	.89	0.01	48.0 (8.1)	47.2 (8.3)	0.9 (−0.4 to 2.2)	.13	0.16
Depression	45.6 (7.6)	45.6 (7.9)	0.1 (−1.0 to 1.2)	.89	0.02	47.6 (7.3)	46.0 (7.5)	1.6 (0.5 to 2.7)	.001	0.34
Pain	47.9 (8.5)	47.8 (8.8)	0.1 (−1.2 to 1.5)	.84	0.02	52.1 (8.2)	50.1 (8.4)	1.9 (0.6 to 3.3)	.001	0.34
Sleep	47.9 (8.5)	48.3 (8.9)	−0.5 (−1.7 to 0.8)	.41	−0.09	49.8 (8.2)	50.5 (8.4)	−0.8 (−2.1 to 0.5)	.16	−0.15
Fatigue	47.0 (7.9)	46.3 (8.2)	0.6 (−0.7 to 1.9)	.28	0.11	48.6 (7.7)	47.4 (7.8)	1.2 (−0.1 to 2.5)	.04	0.22
PCa^h^-specific symptoms: EPIC^i^										
Urinary	82.3 (19.4)	80.7 (20.4)	1.6 (−1.3 to 4.6)	.21	0.14	82.7 (19.0)	84.6 (19.6)	−1.9 (−4.7 to 0.9)	.13	−0.16
Bowel	93.5 (14.3)	92.7 (14.7)	0.8 (−1.7 to 3.2)	.47	0.08	92.6 (13.9)	95.9 (14.0)	−3.2 (−5.7 to −0.8)	.003	−0.31
Sexual	43.7 (31.5)	39.6 (33.3)	4.1 (−0.2 to 8.4)	.03	0.23	50.7 (30.5)	52.0 (31.4)	−1.3 (−5.5 to 3.0)	.51	−0.07
Hormonal	85.0 (23.8)	83.5 (24.8)	1.6 (−2.3 to 5.4)	.36	0.10	79.3 (23.2)	77.6 (23.6)	1.8 (−2.1 to 5.6)	.31	0.11

a The 95% CIs represent Bonferroni-corrected simultaneous CIs for the mean differences between two groups, reported separately for patients and partners. CIs that do not include zero indicate statistically significant differences between groups.

b The *P* values correspond to 2-sided tests of the null hypothesis that the mean difference between 2 groups equals 0. After applying Bonferroni correction for tests conducted separately in patients and partners, a *P* value less than .025 is considered statistically significant.

c Effect sizes (Cohen *d*) are interpreted as small (0.2), medium (0.5), and large (0.8). Effects with |*d*|≥0.5 are considered potentially clinically meaningful.

d QOL: quality of life.

e FACT-G: Functional Assessment of Chronic Illness Therapy-General.

f Higher scores indicated more positive outcomes, ie, better quality of life, better perception of threat of symptoms, less severe symptoms, greater self-efficacy in symptom management, more social support, and better interpersonal support.

g Higher scores indicated more negative outcomes, ie, more frequent or severe symptoms.

h PCa: prostate cancer.

i The EPIC-26 (26-item Expanded Prostate Cancer Index Composite) scores for patients and partners were standardized to enable direct comparison in subsequent analyses.

**Table 6.** Patients’ and partners’ outcomes: time effects by role averaged across study groups for T4 vs T3 (based on multilevel linear mixed models).

Outcome	Patients					Partners				
	T4, mean (SD)	T3, mean (SD)	Difference (95% CI^a^)	*P* value^b^	Effect size^c^	T4, mean (SD)	T3, mean (SD)	Difference (95% CI^a^)	*P* value^b^	Effect size^c^
T4 vs T3										
Primary outcomes: QOL^d^ FACT-G^e^										
FACT-G total score	89.5 (10.4)	90.2 (10.6)	−0.7 (−2.3 to 0.9)	.34	−0.10	86.0 (10.0)	87.8 (10.2)	−1.8 (−3.4 to −0.2)	.01	−0.28
Secondary outcomes: QOL subdomains										
Physical	24.9 (3.3)	24.8 (3.4)	0.1 (−0.4 to 0.6)	.74	0.04	23.8 (3.2)	23.8 (3.3)	−0.1 (−0.6 to 0.5)	.80	−0.03
Social	21.7 (4.1)	22.2 (4.2)	−0.5 (−1.2 to 0.1)	.07	−0.19	21.7 (4.0)	22.2 (4.0)	−0.5 (−1.2 to 0.1)	.06	−0.20
Emotional	20.7 (3.3)	20.7 (3.3)	−0.0 (−0.6 to 0.5)	.85	−0.02	19.9 (3.2)	20.3 (3.2)	−0.4 (−0.9 to 0.1)	.07	−0.19
Functional	22.2 (4.2)	22.3 (4.2)	−0.2 (−0.8 to 0.5)	.53	−0.07	20.6 (4.0)	21.3 (4.1)	−0.7 (−1.3 to −0.1)	.01	−0.26
Secondary outcomes: psychosocial outcomes										
Appraisals^f^										
Appraisal of illness	3.8 (0.6)	3.8 (0.6)	0.0 (−0.1 to 0.1)	.79	0.03	3.8 (0.6)	3.9 (0.6)	−0.1 (−0.2 to 0.0)	.05	−0.21
Coping resources^f^										
Cancer Self-Efficacy Scale	77.3 (11.9)	78.3 (12.1)	−1.0 (−2.9 to 0.9)	.25	−0.13	78.1 (11.5)	78.3 (11.6)	−0.3 (−2.2 to 1.7)	.76	−0.03
Social support^f^										
Emotional support	55.2 (7.0)	55.7 (7.1)	−0.4 (−1.6 to 0.7)	.37	−0.10	53.1 (6.7)	54.0 (6.8)	−0.9 (−2.0 to 0.2)	.07	−0.19
Informational support	56.0 (8.1)	56.6 (8.2)	−0.6 (−2.0 to 0.8)	.34	−0.10	55.5 (7.9)	56.6 (7.9)	−1.2 (−2.5 to 0.2)	.06	−0.20
Instrumental support	58.4 (6.6)	58.7 (6.7)	−0.3 (−1.4 to 0.8)	.53	−0.07	55.9 (6.4)	56.5 (6.4)	−0.5 (−1.6 to 0.6)	.27	−0.12
Secondary outcomes: symptom outcomes										
General symptoms^g^										
Anxiety	46.7 (8.3)	46.4 (8.5)	0.3 (−1.1 to 1.6)	.67	0.05	48.0 (8.1)	47.6 (8.2)	0.4 (−0.9 to 1.7)	.47	0.08
Depression	45.6 (7.6)	45.9 (7.7)	−0.2 (−1.3 to 0.9)	.67	−0.05	47.6 (7.3)	46.1 (7.4)	1.5 (0.4 to 2.6)	.003	0.32
Pain	47.9 (8.5)	48.6 (8.6)	−0.7 (−2.1 to 0.7)	.24	−0.13	52.1 (8.2)	51.2 (8.3)	0.8 (−0.5 to 2.2)	.17	0.15
Sleep	47.9 (8.5)	47.8 (8.7)	0.0 (−1.2 to 1.3)	.95	0.01	49.8 (8.2)	50.3 (8.3)	−0.5 (−1.8 to 0.7)	.34	−0.10
Fatigue	47.0 (7.9)	47.0 (8.1)	−0.0 (−1.3 to 1.3)	.95	−0.01	48.6 (7.7)	48.5 (7.8)	0.0 (−1.3 to 1.3)	.94	0.01
PCa^h^-specific symptoms: EPIC^i^										
Urinary	82.3 (19.4)	83.7 (19.9)	−1.4 (−4.4 to 1.5)	.28	−0.12	82.7 (19.0)	84.8 (16.6)	−2.1 (−5.9 to 1.7)	.21	−0.17
Bowel	93.5 (14.3)	93.5 (14.5)	0.0 (−2.5 to 2.5)	.99	0.00	92.6 (13.9)	94.4 (12.8)	−1.8 (−5.0 to 1.5)	.23	−0.16
Sexual	43.7 (31.5)	41.4 (32.2)	2.3 (−2.1 to 6.6)	.24	0.13	50.7 (30.5)	53.0 (25.6)	−2.3 (−8.1 to 3.4)	.36	−0.13
Hormonal	85.0 (23.8)	84.7 (24.2)	0.4 (−3.5 to 4.3)	.83	0.02	79.3 (23.2)	77.1 (21.0)	2.2 (−2.9 to 7.4)	.33	0.13

a The 95% CIs represent Bonferroni-corrected simultaneous CIs for the mean differences between 2 groups, reported separately for patients and partners. CIs that do not include 0 indicate statistically significant differences between groups.

b The *P* values correspond to 2-sided tests of the null hypothesis that the mean difference between 2 groups equals 0. After applying Bonferroni correction for tests conducted separately in patients and partners, a *P* value less than .025 is considered statistically significant.

c Effect sizes (Cohen *d*) are interpreted as small (0.2), medium (0.5), and large (0.8). Effects with |*d*|≥0.5 are considered potentially clinically meaningful.

d QOL: quality of life.

e FACT-G: Functional Assessment of Chronic Illness Therapy-General.

f Higher scores indicated more positive outcomes, ie, better quality of life, better perception of threat of symptoms, less severe symptoms, greater self-efficacy in symptom management, more social support, and better interpersonal support.

g Higher scores indicated more negative outcomes, ie, more frequent or severe symptoms.

h PCa: prostate cancer.

i The EPIC-26 (26-item Expanded Prostate Cancer Index Composite) scores for patients and partners were standardized to enable direct comparison in subsequent analyses.

Compared with T2, patients reported significantly less severe urinary symptoms at T3 (mean difference 3.0, 95% CI 0.2-5.9; *P*=.02), but lower social well-being scores at T4 (mean difference –0.7, 95% CI –1.4 to –0.1; *P*=.009).

For partners, the effects of time on their functional well-being were significant across all pairwise comparisons (*P*<.001 for T3 vs T2, *P*<.001 for T4 vs T2, and *P*=.02 for T4 vs T3). At T4, partners also reported lower social well-being, lower appraisal of illness, reduced emotional support, reduced instrumental support, increased depression, higher pain severity, and more severe bowel symptoms compared with T2 (*P*<.001, *P*<.001, *P*=.005, *P*=.006, *P*=.001, *P*=.001, and *P*=.003, respectively). Additionally, partners at T4 reported higher depression scores than at T3 (mean difference 1.5, 95% CI 0.4-2.6; *P*=.003).

### Sensitivity Analysis for Missing Data

As shown in the CONSORT flow diagram (Figure 1), some dyads did not complete follow-up assessments across time points due to cancer progression, partner diagnosis with cancer, or loss to follow-up. To assess the robustness of the findings to missing data, sensitivity analyses were conducted by restricting the sample to dyads with complete follow-up data and refitting the same models (Multimedia Appendices 4-6). Overall, the results were broadly consistent with the primary analyses based on all available observations (Tables 1-3). In particular, the conclusions regarding group effects averaged across time for both patients and partners remained unchanged. Some differences were observed in selected time effects and group-by-time comparisons. For example, certain time comparisons for urinary and bowel symptoms, emotional well-being, and illness appraisal differed slightly from those observed in the primary analyses. In addition, several outcomes at T4 reached statistically significant group differences in the complete-case analyses but not in the primary analyses. These differences likely reflect the smaller sample size and selective retention of dyads with complete follow-up.

## Discussion

### Principal Findings

To our knowledge, this is the first fully powered RCT of a theory-guided, couple-focused eHealth intervention to enhance symptom management and improve QOL for patients and their partners managing newly treated PCa. Several design features strengthen the study. Statewide recruitment through the North Carolina Cancer Registry enabled enrollment of a more demographically diverse population than typical single-cancer center trials, helping reduce selection bias. In addition, while most of the trial activities occurred during the COVID-19 pandemic, the digital intervention and flexible research methods, such as phone and Zoom (Zoom Communications, Inc) meetings for intervention and data collection, along with online surveys, allowed the study to continue when most non–COVID-19–related research projects were suspended [34]. The stressful circumstances of the COVID-19 pandemic may have contributed to declines in overall QOL and psychosocial outcomes observed over time for patients and their partner caregivers, underscoring the importance of accessible, digitally delivered interventions such as PERC to support patients and partners during periods of health care disruption. At the same time, the successful completion of the trial demonstrates the feasibility of conducting rigorous psychosocial and educational research under challenging conditions among vulnerable and hard-to-reach populations.

PERC did not yield significant group differences in the primary outcome of QOL (FACT-G total) or most secondary outcomes, including FACT-G subdomain scores and overall psychosocial outcomes. The absence of significant group differences over time suggests that, although PERC was designed to provide informational and social support, it may not have been sufficient to offset broader challenges affecting QOL in this population. Sustaining QOL improvements through short-term interventions is inherently challenging [35], particularly among patients living with prostate cancer who are typically older and often manage multiple comorbid conditions that influence overall health and functioning regardless of intervention exposure [36]. This trial also occurred during the COVID-19 pandemic, which substantially impacted QOL and distress levels among patients living with cancer [37]. Our participants were older adults (mean age 64, SD 6.7 years for patients and mean age 61, SD 7.4 years for partners) managing an average of 4 comorbid conditions, making them particularly vulnerable to pandemic-related stressors and impacts [36]. Increased distress, social isolation, and disruptions in routine care during the study period may have attenuated intervention effects. In addition, anecdotal observations suggest that control participants contacted the research nurse far more frequently than expected during the pandemic. Although these interactions and support primarily addressed COVID-19–related concerns rather than PCa, they may have provided additional support that reduced the observable differences between study groups.

In addition to the limited effects on global QOL, psychosocial outcomes showed minimal between-group differences. This may reflect the complex and multifactorial nature of psychosocial adjustment following cancer treatment, which is influenced by broader life stressors, comorbid health conditions, and caregiving demands that are often difficult to modify through relatively brief supportive interventions [38,39]. Psychosocial adaptation in cancer survivorship also tends to evolve gradually over time and may require more intensive or sustained interventions to produce measurable changes in global outcomes [40]. The modest improvement observed in illness appraisal at 12 months is nevertheless consistent with PERC’s theoretical foundation in stress-coping and dyadic adaptation frameworks, which emphasize cognitive appraisal and coping processes as mechanisms through which individuals adjust to cancer-related stress [16]. Future research may benefit from strengthening intervention intensity, extending follow-up support, or incorporating additional behavioral and caregiver-focused components to enhance effects on broader psychosocial well-being.

Nonetheless, several exploratory but clinically meaningful effects emerged. Patients assigned to the PERC group reported significantly better physical well-being, reduced pain severity, and less frequent fatigue across time, especially at 12 months, compared with the control group. Partners reported less bother about their patients’ urinary symptoms at 8 months. This pattern suggests that the intervention may influence specific symptom domains that are not always reflected in global QOL measures, such as the FACT-G total score. Additionally, global QOL (eg, FACT-G) and psychosocial outcome measures capture broad multidimensional functioning and may be less sensitive to changes in specific symptom-related domains targeted by supportive interventions [41]. While these domain-level trends are encouraging, they must be interpreted as exploratory rather than confirmatory, given the nonsignificant result of the primary outcome. Nonetheless, these findings are noteworthy given the older, geographically diverse population and the added social isolation of the pandemic. This study provides valuable insights for refining future interventions and designing studies powered to detect differences across both global and domain-specific QOL outcomes.

The observed benefits align with PERC’s theoretical foundation, which integrates stress-coping [16] and dyadic adaptation frameworks [42]. By delivering tailored information, interactive skills training, and structured opportunities for joint problem solving, PERC appears to foster supportive behaviors and strengthen symptom management in patients that may help buffer their fatigue and pain. PERC’s interactive format and tailored content may also help facilitate more effective self-management and promote healthier behaviors [43]. Modest but consistent improvements in physical well-being and illness appraisal suggest that even when overall QOL scores remain stable, targeted eHealth support can lessen symptom burden and promote more positive perceptions of the cancer experience. These favorable trends in PERC’s effects emerged during the heightened psychological strain of the pandemic [44], which further underscores the intervention’s relevance and resilience. These findings also mirror a recent systematic review [45] reporting that interventions combining cognitive and educational approaches more effectively improve outcomes than single-component strategies. By integrating both elements, PERC may offer a particularly relevant framework for supporting outcomes such as physical well-being, fatigue, and pain in this population.

Partner caregiver outcomes showed limited and somewhat inconsistent effects. Although no significant group differences were observed in overall QOL or most PCa-specific outcomes for partners, exploratory findings suggested potential benefits in certain areas. For example, partners in the PERC group reported less bother related to patients’ urinary symptoms at T3, indicating that the intervention may have helped partners better manage or interpret specific disease-related concerns. Several factors may explain the limited effects on broader partner outcomes. First, partner caregivers often experience different trajectories of distress and adaptation than patients, with emotional and caregiving burdens emerging or intensifying later in the survivorship period [42,46,47]. Second, interventions that primarily target patient symptom management or dyadic communication may have stronger direct effects on patients than on partners, particularly when caregiver-specific needs such as role strain, emotional burden, and self-care are not addressed explicitly [38,39]. Third, caregiving stress may have been influenced by external contextual factors, such as increased duties and burdens, disruptions to daily routines, health care access, and social support networks during the COVID-19 pandemic [48], which may have attenuated intervention effects on global partner outcomes. Consistent with prior dyadic intervention studies in cancer survivorship, caregiver outcomes often show more modest or variable intervention effects compared with patient outcomes [38,39]. These findings suggest that while PERC may help address specific caregiving concerns, future interventions may benefit from incorporating additional behavioral and caregiver-focused components to achieve consistent improvements in partner well-being and broader psychosocial outcomes.

Our longitudinal findings reveal distinct trajectories of well-being for patients and partners during the year after PCa treatment. Overall QOL declined, with FACT-G total scores significantly lower at 12 months than at 8 months, showing that recovery does not follow a simple upward course. Partners experienced the steepest deterioration, with consistent declines in total and functional QOL, late drops in social well-being and illness appraisal, reduced emotional and instrumental support, and rising depression, pain, and bowel symptom bother. These patterns suggest that caregiving burden and the loss of structured clinical contact accumulate over time, intensifying both emotional and physical strain, effects further compounded by the COVID-19 pandemic [49]. During this period, partner caregivers were simultaneously supporting patients through posttreatment recovery and managing heightened household and family demands, as reported anecdotally by study participants.

In contrast, patients followed a different path: urinary symptoms improved by 8 months, but social well-being declined by 12 months, highlighting late-emerging challenges in relationships and social roles. The findings may relate to the fact that, although PERC was designed to foster mutual support, posttreatment care transitions often prioritize patients’ needs, leaving caregivers’ needs overlooked despite their critical impact on outcomes. High caregiver distress is linked to lower quality of care, greater unplanned health care use, and heavier symptom burdens. Future research should explicitly address caregiver needs and strengthen mutual support between patients and partners.

Notably, many prior RCTs did not evaluate caregiver outcomes [50,51] and those that did reported inconsistent findings [45]. In contrast, this trial systematically assessed both patients and partners across multiple time points, documenting clear time effects for each. These results underscore that survivorship care must extend beyond the immediate posttreatment period and incorporate sustained, targeted caregiver support to preserve QOL and prevent late psychosocial decline.

### Limitations

The COVID-19 pandemic limited access to routine care and heightened distress among older adults with chronic illnesses, which in turn may have influenced the RCT outcomes. The NCI website for the control group underwent substantial improvements during the study, becoming more user-friendly and offering updated and comprehensive supportive care information and resources, and thus, control participants experienced an enhanced standard of care while the support for the PERC group remained consistent. Additionally, control participants also communicated frequently with the research nurse about COVID-19–related concerns and learned strategies for health care and well-being during the stressful time; this additional support may have affected these participants’ stress-coping process and subsequently QOL outcomes. Next, future research should identify subgroups (eg, individuals with limited social support or a high symptom burden) who may benefit the most from PERC and refine the intervention strategies to better mitigate QOL deterioration in survivors of PCa and their caregivers.

The trial participants consisted largely of long-term heterosexual couples with relatively high education and income levels. These characteristics may have facilitated engagement with the eHealth program but may limit generalizability to more diverse populations, including couples with shorter relationship histories, same-sex partners, individuals with lower literacy or digital literacy, and those with limited English proficiency. Future studies should evaluate the intervention in more diverse populations and develop strategies to improve the accessibility and inclusivity of digital supportive care programs.

PERC usage data (eg, module completion and time spent on the platform) were not available for analysis, limiting our ability to examine participants’ engagement with the intervention and its potential influence on outcomes.

Nonetheless, this RCT has several strengths with implications for future research. Despite the challenges of COVID-19, our multidisciplinary team successfully recruited and retained a representative sample using the North Carolina Cancer Registry’s RCA program. We evaluated PERC’s effects on multiple patient and partner outcomes over time, revealing its potential to improve physical well-being, pain, fatigue, and illness appraisals among patients and reduce partners’ bother with patients’ urinary symptoms. Our work also underscores the importance of providing tools and resources to help patients and their families manage health conditions during unexpected socioenvironmental crises caused by natural disasters and pandemics. Our success in recruiting 280 dyads and retaining these participants with high follow-up rates confirms that older adults and their partners can engage with digital health programs when content is accessible and support is available. Such interventions extend the reach of oncology nursing into community and home settings, offering continuous monitoring and support during the critical posttreatment transition when symptoms fluctuate, and caregiver stress is high.

### Conclusion

This RCT evaluated the efficacy of the theory-guided, dyad-focused eHealth PERC program on QOL, symptom management, and coping resources among patient-partner dyads managing newly treated PCa. No significant between-group differences were observed for the primary outcome of overall QOL (FACT-G total score). However, exploratory analyses suggested potential benefits in selected domains: patients in the PERC group reported modest improvements in physical well-being, better appraisal of illness, reduced pain severity at 12 months, and less frequent fatigue across time. PERC partners reported less urinary symptom bother at 8 months. Time effects indicated declines in overall QOL and changes in social and functional well-being over time. Conducted during the COVID-19 pandemic, that is, a period marked by increased distress, social isolation, and disruptions in routine cancer care, these findings suggest that PERC may offer targeted supportive benefits for patients with newly treated localized PCa and their partners, warranting further evaluation in future studies.

## Supplementary material

10.2196/88717 Multimedia Appendix 1 Participant (patients and partners) demographics and characteristics at baseline (T1).

10.2196/88717 Multimedia Appendix 2 Descriptive summaries of outcomes of interest.

10.2196/88717 Multimedia Appendix 3 Estimation results for the fixed effects from the multilevel linear mixed model for the Functional Assessment of Chronic Illness Therapy-General total score.

10.2196/88717 Multimedia Appendix 4 Patients’ and partners’ outcomes: group effects by role averaged across time for dyads with complete follow-up data (based on multilevel linear mixed models).

10.2196/88717 Multimedia Appendix 5 Patients’ and partners’ outcomes: time effects by role averaged across study groups for dyads with complete follow-up data (based on multilevel linear mixed models).

10.2196/88717 Multimedia Appendix 6 Patients’ and partners’ outcomes: group effects by time and role for dyads with complete follow-up data (based on multilevel linear mixed models).

10.2196/88717 Checklist 1 CONSORT checklist.

## Abbreviations

CONSORT: Consolidated Standards of Reporting Trials
EPIC-26: 26-item Expanded Prostate Cancer Index Composite
FACT-G: Functional Assessment of Chronic Illness Therapy-General
MLMM: multilevel linear mixed model
NCI: National Cancer Institute
PCa: prostate cancer
PERC: Prostate Cancer Education and Resources for Couples
PROMIS: Patient-Reported Outcomes Measurement Information System
QOL: quality of life
RCA: rapid case ascertainment
RCT: randomized controlled trial
REDCap: Research Electronic Data Capture
