# Supplementary material for: Evaluating a Tailored Web-Based eHealth Intervention for Symptom Management in Couples Managing Prostate Cancer During the COVID-19 Pandemic: Randomized Clinical Trial
Source: J Med Internet Res. 2026 Jul 10;28:e88717. doi: 10.2196/88717 (PMC13358805; doi:10.2196/88717)
Supplement: Multimedia Appendix 1 [file jmir-v28-e88717-s001.docx]

|  | **Patients** | | | | | | **Partners** | | | | | | | | | | | | |  | | | | |  |
| --- | --- | --- | --- | --- | --- | --- | --- | --- | --- | --- | --- | --- | --- | --- | --- | --- | --- | --- | --- | --- | --- | --- | --- | --- | --- |
|  | **Overall** | | **PERC** | | **Control** | | | **Overall** | | | **PERC** | | | | | **Control** | | | | |  | | | | |
| **Characteristics** | **N** | | **N** | **%** | **N** | **%** | | **N** | **%** | **N** | | **%** | | **N** | | | **%** | | | |  |  |  |  |  |
| **Gender** |  |  |  |  |  |  | |  |  |  | |  | |  | | |  | | | |  |  |  |  |  |
| Male | 280 | | 141 | 50.4 | 139 | 49.6 | | 1 | 0.4 | 0 | | 0 | | 1 | | | 100.0 | | | |  |  |  |  |  |
| Female | - | | - | - | - | - | | 279 | 99.6 | 141 | | 50.5 | | 138 | | | 49.5 | | | |  |  |  |  |  |
| **Race** |  |  |  |  |  |  | |  |  |  | |  | |  | | |  | | | |  |  |  |  |  |
| White | 215 | | 107 | 49.8 | 108 | 50.2 | | 212 | 76.3 | 108 | | 50.9 | | 104 | | | 49.1 | | | |  |  |  |  |  |
| Black | 58 | | 28 | 48.3 | 30 | 51.7 | | 53 | 19.1 | 25 | | 47.2 | | 28 | | | 52.8 | | | |  |  |  |  |  |
| Other | 7 | | 6 | 85.7 | 1 | 14.3 | | 13 | 4.7 | 6 | | 46.2 | | 7 | | | 53.8 | | | |  |  |  |  |  |
| **Ethnicity: Hispanic** |  |  |  |  |  |  | |  |  |  | |  | |  | | |  | | | |  |  |  |  |  |
| No | 270 | | 135 | 50.0 | 135 | 50.0 | | 269 | 97.1 | 135 | | 50.2 | | 134 | | | 49.8 | | | |  |  |  |  |  |
| Yes | 6 | | 4 | 66.7 | 2 | 33.3 | | 8 | 2.9 | 6 | | 75.0 | | 2 | | | 25.0 | | | |  |  |  |  |  |
| **Education** |  |  |  |  |  |  | |  |  |  | |  | |  | | |  | | | |  |  |  |  |  |
| Less than college | 110 | | 52 | 47.3 | 58 | 52.7 | | 111 | 39.6 | 57 | | 51.4 | | 54 | | | 48.6 | | | |  |  |  |  |  |
| Bachelor's degree | 86 | | 40 | 46.5 | 46 | 53.5 | | 83 | 29.6 | 44 | | 53.0 | | 39 | | | 47.0 | | | |  |  |  |  |  |
| Graduate degree (Master's, PhD, JD, MD, etc.) | 66 | | 38 | 57.6 | 28 | 42.4 | | 61 | 21.8 | 28 | | 45.9 | | 33 | | | 54.1 | | | |  |  |  |  |  |
| Other | 17 | | 10 | 58.8 | 7 | 41.2 | | 25 | 8.9 | 12 | | 48.0 | | 13 | | | 52.0 | | | |  |  |  |  |  |
| **Family income** |  |  |  |  |  |  | |  |  |  | |  | |  | | |  | | | |  |  |  |  |  |
| <= $90,000 | 129 | | 72 | 55.8 | 57 | 44.2 | | 122 | 44.0 | 67 | | 54.9 | | 55 | | | 45.1 | | | |  |  |  |  |  |
| > $90,000 | 135 | | 58 | 43.0 | 77 | 57.0 | | 131 | 47.3 | 61 | | 46.6 | | 70 | | | 53.4 | | | |  |  |  |  |  |
| Don't know/refused | 13 | | 9 | 69.2 | 4 | 30.8 | | 24 | 8.7 | 11 | | 45.8 | | 13 | | | 54.2 | | | |  |  |  |  |  |
| **Presently working** |  |  |  |  |  |  | |  |  |  | |  | |  | | |  | | | |  |  |  |  |  |
| No | 127 | | 64 | 50.4 | 63 | 49.6 | | 135 | 48.2 | 63 | | 46.7 | | 72 | | | 53.3 | | | |  |  |  |  |  |
| Yes | 144 | | 70 | 48.6 | 74 | 51.4 | | 140 | 50.0 | 75 | | 53.6 | | 65 | | | 46.4 | | | |  |  |  |  |  |
| Refused to answer | 1 | | 1 | 100.0 | 0 | 0 | | 0 | 0 | - | | - | | - | | | - | | | |  |  |  |  |  |
| Other | 7 | | 5 | 71.4 | 2 | 28.6 | | 5 | 1.8 | 3 | | 60.0 | | 2 | | | 40.0 | | | |  |  |  |  |  |
| **Type of treatment** |  |  |  |  |  |  | |  |  |  | |  | |  | | |  | | | |  |  |  |  |  |
| Surgery | 208 | | 106 | 51.0 | 102 | 49.0 | | - | | | | | | | | | | | | |  | | |  |  |
| Radiation | 72 | | 35 | 48.6 | 37 | 51.4 | |  |  |  |  |  |  |  |  |  |  |  |  |  |  | | |  |  |
| **Gleason score** |  |  |  |  |  |  | |  |  |  | | |  | |  | | |  |  | | | |  |  |  |
| Grade Group 1 | 39 | | 19 | 48.7 | 20 | 51.3 | | - | | | | | | | | | | | | |  | | |  |  |
| Grade Group 2 | 112 | | 56 | 50.0 | 56 | 50.0 | |  |  |  |  |  |  |  |  |  |  |  |  |  |  | | |  |  |
| Grade Group 3 | 71 | | 37 | 52.1 | 34 | 47.9 | |  |  |  |  |  |  |  |  |  |  |  |  |  |  | | |  |  |
| Grade Group 4 | 37 | | 19 | 51.4 | 18 | 48.6 | |  |  |  |  |  |  |  |  |  |  |  |  |  |  | | |  |  |
| Grade Group 5 | 20 | | 9 | 45.0 | 11 | 55.0 | |  |  |  |  |  |  |  |  |  |  |  |  |  |  | | |  |  |
| Mean/SD | **M** | **SD** | **M** | **SD** | **M** | **SD** | | **M** | **SD** | **M** | | | **SD** | | **M** | | | **SD** |  | | | |  |  |  |
| **Age** | 63.7 | 6.7 | 64.3 | 6.9 | 63.1 | 6.6 | | 61.1 | 7.4 | 61.4 | | | 7.2 | | 60.8 | | | 7.5 |  | | | |  |  |  |
| **# Comorbid condition** | 4.2 | 1.9 | 4.1 | 1.8 | 4.3 | 2.0 | | 3.8 | 2.0 | 3.9 | | | 2.0 | | 3.6 | | | 2.0 |  | | | |  |  |  |
| **Prostate-specific antigen** | 2.4 | 1.5 | 2.3 | 1.4 | 2.4 | 1.5 | | - | | | | | | | | | | | | |  | | |  |  |
| **Body mass index** | 28.8 | 4.9 | 29.3 | 5.0 | 28.4 | 4.8 | | 28.3 | 6.2 | 28.3 | | | 5.5 | | 28.3 | | | 6.9 |  | | | |  |  |  |
| **Years of patient-partner relationship** | 32.9 | 12.9 | 33.3 | 12.4 | 32.5 | 13.4 | | 33.2 | 12.8 | 33.6 | | | 12.4 | | 32.9 | | | 13.3 |  | | | |  |  |  |
| **# people supported by income** | 2.4 | 0.9 | 2.4 | 0.9 | 2.4 | 0.9 | | 2.5 | 1.0 | 2.4 | | | 0.9 | | 2.5 | | | 1.1 |  | | | |  |  |  |

**Abbreviations:** PERC: Prostate Cancer Education Resources for Couples; SD: standard deviation.

**Footnote:** Numbers of patient and partner for each variable may vary due to missing data, as some patients and partners did not answer all baseline questions.
