# Supplementary material for: Evaluating a Tailored Web-Based eHealth Intervention for Symptom Management in Couples Managing Prostate Cancer During the COVID-19 Pandemic: Randomized Clinical Trial
Source: J Med Internet Res. 2026 Jul 10;28:e88717. doi: 10.2196/88717 (PMC13358805; doi:10.2196/88717)
Supplement: Multimedia Appendix 2 [file jmir-v28-e88717-s002.docx]

| **Patients** | | | | | | | | | | | | | | | | | | | | | | | | |  |
| --- | --- | --- | --- | --- | --- | --- | --- | --- | --- | --- | --- | --- | --- | --- | --- | --- | --- | --- | --- | --- | --- | --- | --- | --- | --- |
|  | **PERC** | | | | | | | | | | | | **Control** | | | | | | | | | | | |  |
| **Outcomes** | **T1** | | | **T2** | | | **T3** | | | **T4** | | | **T1** | | | **T2** | | | **T3** | | | **T4** | | |  |
|  | **N** | **Mean** | **SD** | **N** | **Mean** | **SD** | **N** | **Mean** | **SD** | **N** | **Mean** | **SD** | **N** | **Mean** | **SD** | **N** | **Mean** | **SD** | **N** | **Mean** | **SD** | **N** | **Mean** | **SD** |  |
| **QOL FACT-G^1^** |  |  |  |  |  |  |  |  |  |  |  |  |  |  |  |  |  |  |  |  |  |  |  |  |  |
| FACT-G total score | 141 | 89.6 | 12.1 | 101 | 91.4 | 12.0 | 86 | 92.0 | 12.6 | 82 | 92.3 | 12.0 | 139 | 88.2 | 15.0 | 102 | 91.2 | 15.0 | 94 | 90.6 | 14.4 | 86 | 89.6 | 16.8 |  |
| **QOL outcomes subdomains^1^** |  |  |  |  |  |  |  |  |  |  |  |  |  |  |  |  |  |  |  |  |  |  |  |  |  |
| Physical | 141 | 24.0 | 3.8 | 102 | 24.7 | 3.9 | 86 | 25.0 | 3.8 | 82 | 25.5 | 3.0 | 139 | 23.7 | 4.5 | 102 | 24.7 | 3.9 | 94 | 24.8 | 4.4 | 86 | 24.8 | 4.2 |  |
| Social | 141 | 22.9 | 3.7 | 101 | 22.8 | 4.0 | 86 | 22.9 | 3.7 | 82 | 22.3 | 4.1 | 139 | 23.1 | 4.2 | 103 | 22.8 | 4.6 | 94 | 22.3 | 4.8 | 86 | 22.0 | 4.7 |  |
| Emotional | 141 | 20.8 | 3.4 | 102 | 21.3 | 3.3 | 86 | 21.2 | 3.5 | 82 | 21.4 | 2.8 | 139 | 20.2 | 4.4 | 103 | 20.8 | 4.2 | 94 | 21.1 | 3.1 | 86 | 20.9 | 4.6 |  |
| Functional | 141 | 21.9 | 4.8 | 102 | 22.4 | 5.2 | 86 | 22.9 | 4.7 | 82 | 23.0 | 4.8 | 139 | 21.2 | 5.4 | 103 | 22.8 | 5.0 | 94 | 22.3 | 5.3 | 86 | 21.9 | 5.9 |  |
| **Appraisals^1^** | |  |  |  |  |  |  |  |  |  |  |  |  |  |  |  |  |  |  |  |  |  |  |  |  |
| Appraisal of illness | | 141 | 3.7 | 0.6 | 101 | 3.9 | 0.7 | 86 | 3.8 | 0.7 | 82 | 3.9 | 0.7 | 139 | 3.8 | 0.7 | 103 | 3.9 | 0.7 | 94 | 3.9 | 0.7 | 86 | 3.8 | 0.7 |
| **Coping resources^1^** | |  |  |  |  |  |  |  |  |  |  |  |  |  |  |  |  |  |  |  |  |  |  |  |  |
| Cancer Self-Efficacy Scale | | 141 | 80.9 | 9.2 | 102 | 80.5 | 10.8 | 86 | 80.7 | 10.4 | 82 | 79.4 | 12.3 | 139 | 80.9 | 9.9 | 103 | 80.5 | 11.2 | 94 | 80.5 | 13.0 | 86 | 79.9 | 12.5 |
| **Social support** | |  |  |  |  |  |  |  |  |  |  |  |  |  |  |  |  |  |  |  |  |  |  |  |  |
| Emotional support | | 141 | 56.5 | 7.3 | 102 | 56.3 | 7.0 | 86 | 57.3 | 7.2 | 82 | 56.5 | 7.9 | 139 | 56.9 | 7.7 | 103 | 56.5 | 7.9 | 94 | 56.7 | 7.5 | 86 | 56.6 | 8.4 |
| Informational support | | 141 | 57.2 | 8.5 | 102 | 57.3 | 7.8 | 86 | 58.3 | 8.4 | 82 | 57.5 | 8.8 | 139 | 57.1 | 7.8 | 103 | 57.0 | 8.9 | 94 | 57.2 | 8.8 | 86 | 57.2 | 9.2 |
| Instrumental support | | 141 | 60.4 | 6.0 | 102 | 60.6 | 5.8 | 86 | 60.3 | 6.4 | 82 | 61.2 | 6.1 | 139 | 60.8 | 5.7 | 103 | 60.8 | 6.1 | 94 | 60.9 | 6.2 | 86 | 59.7 | 7.0 |
| **General symptoms^2^** |  |  |  |  |  |  |  |  |  |  |  |  |  |  |  |  |  |  |  |  |  |  |  |  |  |
| Anxiety | 141 | 45.2 | 8.3 | 102 | 44.5 | 8.1 | 86 | 44.3 | 8.5 | 82 | 44.1 | 8.0 | 139 | 46.1 | 9.4 | 103 | 45.1 | 9.0 | 94 | 44.9 | 9.2 | 86 | 45.4 | 10.4 |  |
| Depression | 141 | 44.7 | 7.9 | 102 | 44.1 | 7.5 | 86 | 44.8 | 7.8 | 82 | 43.5 | 7.7 | 139 | 45.0 | 9.1 | 103 | 44.2 | 8.8 | 94 | 44.4 | 8.7 | 86 | 44.8 | 9.3 |  |
| Pain | 141 | 48.3 | 8.7 | 102 | 47.2 | 8.9 | 86 | 47.3 | 8.7 | 82 | 45.9 | 6.9 | 139 | 48.5 | 9.1 | 103 | 46.4 | 7.8 | 94 | 48.2 | 9.6 | 86 | 47.8 | 8.8 |  |
| Sleep | 141 | 48.9 | 9.4 | 102 | 47.3 | 9.3 | 86 | 47.3 | 8.6 | 82 | 46.9 | 8.5 | 139 | 49.9 | 9.5 | 103 | 48.9 | 9.4 | 94 | 48.5 | 9.2 | 86 | 49.1 | 9.8 |  |
| Fatigue | 141 | 46.1 | 9.4 | 102 | 44.3 | 9.8 | 86 | 45.6 | 9.9 | 82 | 43.8 | 8.9 | 139 | 47.3 | 9.4 | 103 | 46.4 | 9.9 | 94 | 46.8 | 9.6 | 86 | 47.8 | 9.1 |  |
| **PCa-specific symptoms: EPIC^1,3^** |  |  |  |  |  |  |  |  |  |  |  |  |  |  |  |  |  |  |  |  |  |  |  |  |  |
| Urinary | 137 | 67.5 | 20.5 | 102 | 79.3 | 16.4 | 82 | 82.6 | 16.2 | 78 | 80.6 | 16.0 | 134 | 68.1 | 19.3 | 101 | 79.2 | 16.3 | 94 | 81.0 | 14.7 | 85 | 80.7 | 17.5 |  |
| Bowel | 141 | 91.9 | 14.2 | 103 | 93.9 | 14.8 | 87 | 94.2 | 10.8 | 81 | 95.2 | 10.0 | 139 | 90.0 | 13.9 | 101 | 93.2 | 12.7 | 94 | 93.6 | 13.5 | 86 | 92.4 | 13.0 |  |
| Sexual | 137 | 23.0 | 24.1 | 101 | 25.0 | 26.7 | 86 | 27.6 | 26.3 | 80 | 28.2 | 29.4 | 134 | 21.5 | 25.6 | 97 | 27.4 | 27.1 | 87 | 27.1 | 24.4 | 85 | 32.1 | 26.6 |  |
| Hormonal | 141 | 87.6 | 14.2 | 103 | 85.2 | 18.1 | 86 | 86.7 | 13.9 | 82 | 89.4 | 11.2 | 138 | 85.6 | 15.7 | 103 | 87.4 | 14.5 | 94 | 87.0 | 15.0 | 86 | 86.1 | 16.2 |  |
| **Partners** | | | | | | | | | | | | | | | | | | | | | | | | |  |
|  | **PERC** | | | | | | | | | | | | **Control** | | | | | | | | | | | |  |
| **Outcomes** | **T1** | | | **T2** | | | **T3** | | | **T4** | | | **T1** | | | **T2** | | | **T3** | | | **T4** | | |  |
|  | **N** | **Mean** | **SD** | **N** | **Mean** | **SD** | **N** | **Mean** | **SD** | **N** | **Mean** | **SD** | **N** | **Mean** | **SD** | **N** | **Mean** | **SD** | **N** | **Mean** | **SD** | **N** | **Mean** | **SD** |  |
| **QOL FACT-G^1^** |  |  |  |  |  |  |  |  |  |  |  |  |  |  |  |  |  |  |  |  |  |  |  |  |  |
| FACT-G total score | 140 | 89.4 | 13.7 | 102 | 89.7 | 13.9 | 84 | 88.5 | 16.1 | 79 | 88.6 | 13.0 | 139 | 88.6 | 14.7 | 99 | 89.2 | 15.8 | 93 | 87.5 | 16.3 | 88 | 86.5 | 16.9 |  |
| **QOL outcomes subdomains^1^** |  |  |  |  |  |  |  |  |  |  |  |  |  |  |  |  |  |  |  |  |  |  |  |  |  |
| Physical | 141 | 24.7 | 3.6 | 102 | 24.5 | 3.6 | 84 | 24.4 | 4.0 | 79 | 24.8 | 3.3 | 139 | 24.1 | 4.7 | 99 | 24.0 | 4.7 | 93 | 23.9 | 4.7 | 88 | 23.8 | 4.7 |  |
| Social | 141 | 22.9 | 5.0 | 102 | 23.0 | 4.6 | 84 | 22.3 | 5.1 | 79 | 22.0 | 4.6 | 139 | 23.0 | 4.1 | 99 | 22.6 | 4.6 | 93 | 22.1 | 4.9 | 88 | 22.0 | 4.9 |  |
| Emotional | 141 | 19.9 | 3.7 | 102 | 20.2 | 3.7 | 84 | 20.2 | 4.2 | 79 | 20.3 | 3.3 | 139 | 19.6 | 4.1 | 99 | 20.2 | 4.1 | 93 | 20.2 | 4.1 | 88 | 19.9 | 4.3 |  |
| Functional | 141 | 21.8 | 4.6 | 102 | 22.0 | 4.8 | 84 | 21.6 | 5.4 | 79 | 21.5 | 4.6 | 139 | 21.9 | 5.0 | 99 | 22.5 | 4.9 | 93 | 21.3 | 5.4 | 88 | 20.8 | 5.9 |  |
| **Appraisals^1^** |  |  |  |  |  |  |  |  |  |  |  |  |  |  |  |  |  |  |  |  |  |  |  |  |  |
| Appraisal of illness | 140 | 3.8 | 0.6 | 102 | 3.9 | 0.6 | 84 | 3.9 | 0.7 | 80 | 3.9 | 0.6 | 139 | 3.8 | 0.7 | 99 | 4.0 | 0.7 | 93 | 3.9 | 0.7 | 88 | 3.8 | 0.7 |  |
| **Coping resources^1^** |  |  |  |  |  |  |  |  |  |  |  |  |  |  |  |  |  |  |  |  |  |  |  |  |  |
| Cancer Self-Efficacy Scale | 141 | 76.9 | 12.4 | 102 | 78.7 | 12.1 | 84 | 76.6 | 13.6 | 79 | 78.5 | 11.2 | 139 | 80.4 | 10.4 | 99 | 78.7 | 12.8 | 93 | 79.9 | 11.7 | 88 | 79.1 | 13.1 |  |
| **Social support** |  |  |  |  |  |  |  |  |  |  |  |  |  |  |  |  |  |  |  |  |  |  |  |  |  |
| Emotional support | 141 | 55.5 | 8.4 | 102 | 54.5 | 8.1 | 84 | 53.7 | 8.2 | 79 | 54.1 | 8.3 | 139 | 54.9 | 7.4 | 99 | 54.7 | 7.0 | 93 | 54.7 | 7.9 | 87 | 53.3 | 8.5 |  |
| Informational support | 140 | 56.5 | 9.3 | 102 | 56.8 | 9.4 | 84 | 57.6 | 9.0 | 79 | 57.1 | 8.6 | 139 | 56.7 | 8.4 | 99 | 56.1 | 7.6 | 93 | 56.8 | 9.1 | 88 | 56.2 | 9.2 |  |
| Instrumental support | 141 | 56.1 | 8.5 | 102 | 56.9 | 8.1 | 84 | 54.8 | 8.9 | 79 | 54.7 | 8.4 | 139 | 55.5 | 8.1 | 99 | 55.5 | 7.6 | 93 | 55.5 | 8.5 | 88 | 55.3 | 9.0 |  |
| **General symptoms^2^** |  |  |  |  |  |  |  |  |  |  |  |  |  |  |  |  |  |  |  |  |  |  |  |  |  |
| Anxiety | 141 | 49.0 | 9.5 | 102 | 48.0 | 9.2 | 84 | 48.5 | 9.2 | 79 | 46.8 | 9.0 | 139 | 49.5 | 9.8 | 99 | 48.2 | 10.7 | 93 | 48.7 | 10.3 | 88 | 49.6 | 9.7 |  |
| Depression | 141 | 46.2 | 8.7 | 102 | 46.4 | 9.2 | 84 | 46.8 | 9.2 | 79 | 46.6 | 7.8 | 139 | 46.3 | 8.9 | 99 | 45.7 | 9.0 | 93 | 45.4 | 9.0 | 88 | 47.2 | 9.3 |  |
| Pain | 141 | 49.5 | 9.4 | 102 | 49.5 | 9.1 | 84 | 51.2 | 9.6 | 79 | 51.0 | 8.3 | 139 | 50.8 | 9.9 | 99 | 50.8 | 9.8 | 93 | 52.0 | 10.7 | 88 | 52.8 | 10.1 |  |
| Sleep | 141 | 48.7 | 10.0 | 102 | 49.8 | 9.0 | 84 | 49.9 | 9.8 | 79 | 48.0 | 9.8 | 139 | 50.0 | 9.5 | 99 | 51.3 | 11.0 | 93 | 50.7 | 10.4 | 88 | 50.5 | 10.3 |  |
| Fatigue | 141 | 47.9 | 9.4 | 102 | 47.3 | 9.2 | 84 | 48.5 | 9.9 | 79 | 47.8 | 8.8 | 139 | 49.5 | 9.7 | 99 | 48.4 | 9.6 | 93 | 50.4 | 10.1 | 88 | 50.0 | 9.3 |  |
| **PCa-specific symptoms: EPIC^1,3^** |  |  |  |  |  |  |  |  |  |  |  |  |  |  |  |  |  |  |  |  |  |  |  |  |  |
| Urinary | 141 | 77.0 | 30.2 | 102 | 86.0 | 21.3 | 34 | 90.4 | 17.4 | 80 | 86.9 | 23.2 | 139 | 78.6 | 26.5 | 101 | 87.6 | 19.9 | 47 | 86.7 | 26.0 | 87 | 83.0 | 26.3 |  |
| Bowel | 141 | 91.1 | 20.7 | 101 | 96.8 | 9.8 | 34 | 97.8 | 9.5 | 80 | 95.0 | 13.4 | 139 | 92.4 | 16.9 | 100 | 96.0 | 14.1 | 47 | 94.1 | 17.5 | 88 | 92.0 | 17.6 |  |
| Sexual | 139 | 65.1 | 38.3 | 99 | 66.2 | 36.1 | 32 | 64.1 | 34.7 | 80 | 61.6 | 36.4 | 138 | 63.2 | 39.6 | 99 | 60.9 | 37.8 | 45 | 66.7 | 38.1 | 87 | 65.2 | 36.2 |  |
| Hormonal | 141 | 76.4 | 30.3 | 101 | 77.2 | 31.0 | 34 | 73.5 | 37.9 | 80 | 75.3 | 31.4 | 139 | 77.0 | 28.6 | 100 | 73.8 | 32.1 | 47 | 73.4 | 33.9 | 88 | 80.4 | 27.2 |  |

**Abbreviation:** QOL: quality of life; FACT-G: Functional Assessment of Chronic Illness Therapy-General; PCa: prostate cancer; EPIC, Expanded Prostate Cancer Index Composite; PERC: Prostate Cancer Education Resources for Couples.

**Footnote:**

1.Higher scores indicated more positive outcomes: ie, better quality of life, better perception of threat of symptoms, less severe symptoms, greater self-efficacy in symptom management, more social support, and better interpersonal support.

2.Higher scores indicated more negative outcomes: ie, more severe symptoms.

3.The EPIC-26 (26-item Expanded Prostate Cancer Index Composite) scores for patients and partners were standardized to enable direct comparison in subsequent analyses.
