# Supplementary material for: Evaluating a Tailored Web-Based eHealth Intervention for Symptom Management in Couples Managing Prostate Cancer During the COVID-19 Pandemic: Randomized Clinical Trial
Source: J Med Internet Res. 2026 Jul 10;28:e88717. doi: 10.2196/88717 (PMC13358805; doi:10.2196/88717)
Supplement: Multimedia Appendix 3 [file jmir-v28-e88717-s003.docx]

| **Fixed effect** | **Estimate** | **SE** | ***P* value** |
| --- | --- | --- | --- |
| Intercept | 17.32 | 2.56 | <.001 |
| FACT-G total score at T1 | 0.81 | 0.03 | <.001 |
| Group (referent: PERC) | -0.10 | 1.27 | .94 |
| **Time point (referent: T2)** |  |  |  |
| T3 | -1.65 | 0.95 | .08 |
| T4 | -3.67 | 0.97 | <.001 |
| Role (referent: patient) | 1.12 | 1.22 | .36 |
| **Family income (referent: <= $90,000)** |  |  |  |
| > $90,000 | 0.85 | 0.80 | .29 |
| Don't know/refused | -2.45 | 1.55 | .11 |
| ***Interaction Terms*** |  |  |  |
| Group × Time point T3 | 0.13 | 1.36 | .92 |
| Group × Time point T4 | 0.58 | 1.39 | .68 |
| Group × Role | -0.50 | 1.73 | .77 |
| Time point T3 × Role | 0.37 | 1.34 | .78 |
| Time point T4 × Role | 1.50 | 1.38 | .27 |
| Group × Time point T3 × Role | 2.32 | 1.92 | .23 |
| Group × Time point T4 × Role | 2.29 | 1.97 | .24 |

**Abbreviation:** FACT-G: Functional Assessment of Chronic Illness Therapy-General; PERC: Prostate Cancer Education Resources for Couples; SE: standard error.
