# Supplementary material for: Evaluating a Tailored Web-Based eHealth Intervention for Symptom Management in Couples Managing Prostate Cancer During the COVID-19 Pandemic: Randomized Clinical Trial
Source: J Med Internet Res. 2026 Jul 10;28:e88717. doi: 10.2196/88717 (PMC13358805; doi:10.2196/88717)
Supplement: Multimedia Appendix 4 [file jmir-v28-e88717-s004.docx]

|  | **Patients** | | | | | **Partners** | | | | | |
| --- | --- | --- | --- | --- | --- | --- | --- | --- | --- | --- | --- |
|  | **Mean (SD)** | |  |  |  | **Mean (SD)** | | |  |  |  |
|  | **PERC** | **Control** | **Difference**  **(95% CI^4^)** | ***P* value^5^** | **Effect**  **size^6^** | **PERC** | **Control** | **Difference**  **(95% CI^4^)** | | ***P* value^5^** | **Effect size^6^** |
| **Primary outcomes**  **QOL FACT-G^1^** |  |  |  |  |  |  |  | |  |  |  |
| FACT-G total score | 90.8 (14.9) | 88.1 (15.4) | 2.7 (-0.2, 5.6) | .04 | 0.20 | 88.2 (14.9) | 87.9 (14.5) | | 0.3 (-2.6, 3.2) | .84 | 0.02 |
| **Secondary outcomes:** |  |  |  |  |  |  |  | |  |  |  |
| **QOL outcomes subdomains^1^** |  |  |  |  |  |  |  | |  |  |  |
| Physical | 24.9 (4.8) | 24.1 (4.9) | 0.7 (-0.2, 1.7) | .07 | 0.18 | 23.7 (4.8) | 23.6 (4.6) | | 0.1 (-0.8, 1.1) | .75 | 0.03 |
| Social | 22.5 (5.5) | 21.8 (5.7) | 0.7 (-0.3, 1.8) | .13 | 0.15 | 22.7 (5.5) | 22.2 (5.3) | | 0.6 (-0.5, 1.6) | .23 | 0.12 |
| Emotional | 20.7 (4.2) | 20.3 (4.3) | 0.4 (-0.4, 1.2) | .28 | 0.11 | 20.2 (4.2) | 20.4 (4.1) | | -0.2 (-1.0, 0.6) | .58 | -0.05 |
| Functional | 22.5 (6.0) | 21.9 (6.2) | 0.6 (-0.5, 1.8) | .22 | 0.12 | 21.5 (6.0) | 21.5 (5.8) | | -0.0 (-1.2, 1.1) | .94 | -0.01 |
| **Secondary outcomes: Psychosocial outcomes** |  |  |  |  |  |  |  | |  |  |  |
| **Appraisals^1^** |  |  |  |  |  |  |  | |  |  |  |
| Appraisal of illness | 3.9 (0.8) | 3.8 (0.8) | 0.1 (-0.0, 0.3) | .04 | 0.20 | 3.9 (0.8) | 3.9 (0.8) | | 0.0 (-0.1, 0.2) | .79 | 0.03 |
| **Coping resources^1^** |  |  |  |  |  |  |  | |  |  |  |
| Cancer Self-Efficacy Scale | 78.3 (14.9) | 78.2 (15.4) | 0.1 (-2.7, 3.0) | .91 | 0.01 | 78.6 (15.0) | 78.5 (14.4) | | 0.2 (-2.7, 3.1) | .89 | 0.01 |
| **Social support^1^** |  |  |  |  |  |  |  | |  |  |  |
| Emotional support | 55.9 (9.3) | 54.8 (9.6) | 1.2 (-0.6, 3.0) | .15 | 0.14 | 53.5 (9.3) | 53.8 (9.0) | | -0.3 (-2.1, 1.5) | .71 | -0.04 |
| Informational support | 56.3 (9.9) | 56.2 (10.2) | 0.1 (-1.8, 2.0) | .89 | 0.01 | 56.8 (9.9) | 56.0 (9.6) | | 0.8 (-1.1, 2.7) | .34 | 0.09 |
| Instrumental support | 58.9 (9.0) | 58.1 (9.3) | 0.8 (-0.9, 2.5) | .31 | 0.10 | 56.3 (9.0) | 56.5 (8.7) | | -0.2 (-1.9, 1.6) | .84 | -0.02 |
| **Secondary outcomes: Symptom outcomes** |  |  |  |  |  |  |  | |  |  |  |
| **General symptoms**^2^ |  |  |  |  |  |  |  | |  |  |  |
| Anxiety | 46.9 (11.2) | 47.0 (11.4) | -0.1 (-2.2, 2.1) | .94 | -0.01 | 47.2 (11.1) | 46.7 (10.9) | | 0.5 (-1.7, 2.6) | .62 | 0.05 |
| Depression | 45.9 (10.4) | 46.1 (10.7) | -0.2 (-2.2, 1.8) | .80 | -0.02 | 46.4 (10.4) | 45.5 (10.1) | | 0.8 (-1.2, 2.9) | .34 | 0.09 |
| Pain | 47.4 (11.8) | 48.9 (12.2) | -1.5 (-3.8, 0.7) | .13 | -0.15 | 51.0 (11.8) | 51.7 (11.6) | | -0.7 (-3.0, 1.6) | .50 | -0.07 |
| Sleep | 47.4 (12.5) | 49.2 (12.9) | -1.8 (-4.2, 0.6) | .10 | -0.16 | 50.2 (12.5) | 50.3 (12.1) | | -0.1 (-2.6, 2.3) | .90 | -0.01 |
| Fatigue | 45.5 (11.0) | 47.7 (11.3) | -2.2 (-4.3, -0.1) | .02 | -0.23 | 47.5 (11.0) | 47.9 (10.8) | | -0.3 (-2.5, 1.8) | .73 | -0.03 |
| **PCa-specific symptoms: EPIC ^1,3^** |  |  |  |  |  |  |  | |  |  |  |
| Urinary | 86.2 (26.6) | 84.3 (27.5) | 1.9 (-6.0, 9.8) | .58 | 0.08 | 87.9 (25.8) | 86.8 (27.0) | | 1.1 (-6.9, 9.1) | .75 | 0.05 |
| Bowel | 96.7 (17.9) | 93.0 (18.9) | 3.7 (-1.6, 9.1) | .12 | 0.23 | 95.4 (17.6) | 96.5 (17.9) | | -1.2 (-6.6, 4.2) | .62 | -0.07 |
| Sexual | 46.5 (53.5) | 43.8 (56.8) | 2.6 (-11.1, 16.4) | .66 | 0.07 | 61.3 (49.5) | 62.8 (51.2) | | -1.5 (-15.3,12.3) | .80 | -0.04 |
| Hormonal | 84.3 (31.4) | 84.7 (32.3) | -0.5 (-9.6, 8.6) | .90 | -0.02 | 80.4 (30.6) | 80.5 (30.8) | | -0.1 (-9.3, 9.1) | .98 | -0.00 |

**Abbreviation:** QOL: quality of life; FACT-G: Functional Assessment of Chronic Illness Therapy-General; PCa: prostate cancer; EPIC, Expanded Prostate Cancer Index Composite; PERC: Prostate Cancer Education Resources for Couples.

**Footnote:**

1.Higher scores indicated more positive outcomes: ie, better quality of life, better perception of threat of symptoms, less severe symptoms, greater self-efficacy in symptom management, more social support, and better interpersonal support.

2.Higher scores indicated more negative outcomes: ie, more frequent or severe symptoms.

3.The EPIC-26 (26-item Expanded Prostate Cancer Index Composite) scores for patients and partners were standardized to enable direct comparison in subsequent analyses.

4. The 95% CIs represent Bonferroni-corrected simultaneous CIs for the mean differences between two groups, reported separately for patients and partners. CIs that do not include zero indicate statistically significant differences between groups.

5. The *P* values correspond to 2-sided tests of the null hypothesis that the mean difference between two groups equals zero. After applying Bonferroni correction for tests conducted separately in patients and partners, a *P* value less than 0.025 is considered statistically significant.

6. Effect sizes (Cohen *d*) are interpreted as small (0.2), medium (0.5), and large (0.8). Effects with |*d*|≥0.5 are considered potentially clinically meaningful.
