# Supplementary material for: Evaluating a Tailored Web-Based eHealth Intervention for Symptom Management in Couples Managing Prostate Cancer During the COVID-19 Pandemic: Randomized Clinical Trial
Source: J Med Internet Res. 2026 Jul 10;28:e88717. doi: 10.2196/88717 (PMC13358805; doi:10.2196/88717)
Supplement: Multimedia Appendix 5 [file jmir-v28-e88717-s005.docx]

|  | **Patients** | | | | | **Partners** | | | | |
| --- | --- | --- | --- | --- | --- | --- | --- | --- | --- | --- |
|  | **Mean (SD)** | | **Difference**  **(95% CI^4^)** |  |  | **Mean (SD)** | | **Difference**  **(95% CI^4^)** |  |  |
|  | **Time point 1** | **Time point**  **2** |  | ***P* value^5^** | **Effect size^6^** | **Time point 1** | **Time point 2** |  | ***P* value^5^** | **Effect size^6^** |
| **T3 vs. T2** | **T3** | **T2** |  |  |  | **T3** | **T2** |  |  |  |
| **Primary outcomes**  **QOL FACT-G^1^** |  |  |  |  |  |  |  |  |  |  |
| FACT-G total score | 89.3 (11.1) | 89.8 (11.1) | -0.5 (-2.3, 1.2) | .49 | -0.08 | 88.3 (10.7) | 90.0 (10.7) | -1.7 (-3.5, 0.0) | .03 | -0.27 |
| **Secondary outcomes:** |  |  |  |  |  |  |  |  |  |  |
| **QOL subdomains^1^** |  |  |  |  |  |  |  |  |  |  |
| Physical | 24.5 (3.6) | 24.3 (3.6) | 0.2 (-0.4, 0.8) | .46 | 0.09 | 23.7 (3.4) | 23.8 (3.4) | -0.1 (-0.7, 0.4) | .64 | -0.06 |
| Social | 22.1 (4.2) | 22.5 (4.2) | -0.4 (-1.1, 0.3) | .21 | -0.15 | 22.5 (4.0) | 23.1 (4.0) | -0.6 (-1.3, 0.1) | .04 | -0.25 |
| Emotional | 20.5 (3.3) | 20.5 (3.3) | -0.0 (-0.6, 0.6) | .98 | -0.00 | 20.5 (3.1) | 20.4 (3.1) | 0.1 (-0.5, 0.7) | .71 | 0.05 |
| Functional | 22.1 (4.5) | 22.3 (4.5) | -0.2 (-0.9, 0.5) | .45 | -0.09 | 21.4 (4.3) | 22.5 (4.3) | -1.1 (-1.8, -0.4) | <.001 | -0.44 |
| **Secondary outcomes: Psychosocial outcomes** |  |  |  |  |  |  |  |  |  |  |
| **Appraisals^1^** |  |  |  |  |  |  |  |  |  |  |
| Appraisal of illness | 3.8 (0.6) | 3.9 (0.6) | -0.1 (-0.2, 0.0) | .12 | -0.19 | 3.9 (0.6) | 3.9 (0.6) | -0.0 (-0.1, 0.1) | .84 | -0.02 |
| **Coping resources^1^** |  |  |  |  |  |  |  |  |  |  |
| Cancer Self-Efficacy Scale | 77.8 (11.6) | 79.4 (11.6) | -1.6 (-3.6, 0.5) | .08 | -0.21 | 78.6 (11.2) | 79.1 (11.2) | -0.4 (-2.5, 1.6) | .63 | -0.06 |
| **Social support^1^** |  |  |  |  |  |  |  |  |  |  |
| Emotional support | 55.5 (7.1) | 55.2 (7.1) | 0.4 (-0.8, 1.6) | .48 | 0.08 | 53.7 (6.9) | 54.3 (6.9) | -0.6 (-1.8, 0.6) | .29 | -0.13 |
| Informational support | 56.8 (7.9) | 55.8 (7.9) | 1.0 (-0.5, 2.5) | .13 | 0.18 | 57.2 (7.6) | 56.2 (7.6) | 1.0 (-0.4, 2.5) | .12 | 0.19 |
| Instrumental support | 58.6 (7.0) | 58.4 (7.0) | 0.1 (-1.0, 1.3) | .79 | 0.03 | 56.6 (6.7) | 57.0 (6.7) | -0.4 (-1.6, 0.8) | .46 | -0.09 |
| **Secondary outcomes: Symptom outcomes** |  |  |  |  |  |  |  |  |  |  |
| **General symptoms**^2^ |  |  |  |  |  |  |  |  |  |  |
| Anxiety | 47.0 (8.5) | 46.9 (8.5) | 0.1 (-1.4, 1.5) | .93 | 0.01 | 46.9 (8.2) | 46.6 (8.2) | 0.3 (-1.1, 1.7) | .62 | 0.06 |
| Depression | 46.4 (7.7) | 45.9 (7.7) | 0.5 (-0.7, 1.7) | .38 | 0.11 | 45.5 (7.5) | 45.5 (7.5) | 0.1 (-1.1, 1.3) | .92 | 0.01 |
| Pain | 48.5 (8.9) | 48.0 (8.9) | 0.5 (-0.9, 2.0) | .43 | 0.10 | 51.4 (8.7) | 50.3 (8.7) | 1.1 (-0.4, 2.5) | .09 | 0.20 |
| Sleep | 48.1 (9.2) | 48.9 (9.2) | -0.8 (-2.1, 0.6) | .20 | -0.15 | 50.2 (8.9) | 50.7 (8.9) | -0.5 (-1.9, 0.8) | .37 | -0.11 |
| Fatigue | 47.1 (8.4) | 46.1 (8.4) | 1.0 (-0.4, 2.5) | .10 | 0.20 | 48.0 (8.2) | 46.9 (8.2) | 1.1 (-0.3, 2.6) | .07 | 0.22 |
| **PCa-specific symptoms: EPIC ^1,3^** |  |  |  |  |  |  |  |  |  |  |
| Urinary | 87.1 (20.0) | 83.6 (20.0) | 3.5 (-1.3, 8.2) | .10 | 0.30 | 88.8 (19.2) | 87.8 (19.2) | 1.0 (-3.7, 5.8) | .62 | 0.09 |
| Bowel | 95.2 (14.7) | 92.4 (14.7) | 2.8 (-1.4, 7.0) | .14 | 0.26 | 96.4 (14.1) | 97.4 (14.1) | -1.0 (-5.3, 3.2) | .58 | -0.10 |
| Sexual | 44.0 (42.1) | 44.8 (42.1) | -0.8 (-8.6, 6.9) | .81 | -0.05 | 64.0 (37.8) | 62.2 (37.8) | 1.7 (-6.0, 9.5) | .61 | 0.10 |
| Hormonal | 84.9 (25.0) | 83.6 (25.0) | 1.3 (-5.6, 8.2) | .67 | 0.08 | 81.6 (24.0) | 78.8 (24.0) | 2.8 (-4.1, 9.7) | .36 | 0.16 |
|  | **Patients** | | | | | **Partners** | | | | |
|  | **Mean (SD)** | |  |  |  | **Mean (SD)** | |  |  |  |
|  | **Time point 1** | **Time point**  **2** | **Difference**  **(95% CI^4^)** | ***P* value^5^** | **Effect size^6^** | **Time point 1** | **Time point 2** | **Difference**  **(95% CI^4^)** | ***P* value^5^** | **Effect size^6^** |
| **T4 vs. T2** | **T4** | **T2** |  |  |  | **T4** | **T2** |  |  |  |
| **Primary outcomes**  **QOL^1^ FACT-G** |  |  |  |  |  |  |  |  |  |  |
| FACT-G total score | 89.3 (11.1) | 89.8 (11.1) | -0.4 (-2.2, 1.3) | .56 | -0.07 | 85.9 (10.7) | 90.0 (10.7) | -4.1 (-5.9, -2.4) | <.001 | -0.65 |
| **Secondary outcomes:** |  |  |  |  |  |  |  |  |  |  |
| **QOL subdomains** |  |  |  |  |  |  |  |  |  |  |
| Physical | 24.7 (3.6) | 24.3 (3.6) | 0.3 (-0.2, 0.9) | .19 | 0.16 | 23.5 (3.4) | 23.8 (3.4) | -0.3 (-0.9, 0.3) | .23 | -0.15 |
| Social | 21.8 (4.2) | 22.5 (4.2) | -0.7 (-1.4, -0.1) | .02 | -0.29 | 21.7 (4.0) | 23.1 (4.0) | -1.4 (-2.0, -0.7) | <.001 | -0.54 |
| Emotional | 20.5 (3.3) | 20.5 (3.3) | -0.0 (-0.6, 0.6) | .95 | -0.01 | 19.9 (3.1) | 20.4 (3.1) | -0.5 (-1.0, 0.1) | .07 | -0.22 |
| Functional | 22.2 (4.5) | 22.3 (4.5) | -0.0 (-0.7, 0.6) | .89 | -0.02 | 20.6 (4.3) | 22.5 (4.3) | -2.0 (-2.6, -1.3) | <.001 | -0.78 |
| **Secondary outcomes: Psychosocial outcomes** |  |  |  |  |  |  |  |  |  |  |
| **Appraisals^1^** |  |  |  |  |  |  |  |  |  |  |
| Appraisal of illness | 3.8 (0.6) | 3.9 (0.6) | -0.0 (-0.1, 0.1) | .36 | -0.11 | 3.8 (0.6) | 3.9 (0.6) | -0.1 (-0.3, -0.0) | .002 | -0.38 |
| **Coping resources^1^** |  |  |  |  |  |  |  |  |  |  |
| Cancer Self-Efficacy Scale | 77.6 (11.6) | 79.4 (11.6) | -1.8 (-3.8, 0.2) | .05 | -0.24 | 78.0 (11.2) | 79.1 (11.2) | -1.1 (-3.1, 0.9) | .22 | -0.15 |
| **Social support^1^** |  |  |  |  |  |  |  |  |  |  |
| Emotional support | 55.4 (7.1) | 55.2 (7.1) | 0.2 (-1.0, 1.4) | .66 | 0.05 | 53.0 (6.9) | 54.3 (6.9) | -1.3 (-2.5, -0.1) | .02 | -0.28 |
| Informational support | 56.1 (7.9) | 55.8 (7.9) | 0.4 (-1.1, 1.8) | .56 | 0.07 | 55.8 (7.6) | 56.2 (7.6) | -0.4 (-1.8, 1.1) | .57 | -0.07 |
| Instrumental support | 58.4 (7.0) | 58.4 (7.0) | -0.0 (-1.2, 1.2) | .99 | -0.00 | 55.7 (6.7) | 57.0 (6.7) | -1.3 (-2.5, -0.2) | .01 | -0.31 |
| **Secondary outcomes: Symptom outcomes** |  |  |  |  |  |  |  |  |  |  |
| **General symptoms**^2^ |  |  |  |  |  |  |  |  |  |  |
| Anxiety | 46.9 (8.5) | 46.9 (8.5) | -0.0 (-1.5, 1.4) | .95 | -0.01 | 47.4 (8.2) | 46.6 (8.2) | 0.8 (-0.6, 2.2) | .20 | 0.15 |
| Depression | 45.7 (7.7) | 45.9 (7.7) | -0.3 (-1.4, 0.9) | .63 | -0.06 | 46.9 (7.5) | 45.5 (7.5) | 1.5 (0.3, 2.7) | .005 | 0.34 |
| Pain | 48.0 (8.9) | 48.0 (8.9) | -0.1 (-1.5, 1.4) | .91 | -0.01 | 52.5 (8.7) | 50.3 (8.7) | 2.2 (0.7, 3.6) | <.001 | 0.41 |
| Sleep | 47.9 (9.2) | 48.9 (9.2) | -1.0 (-2.4, 0.3) | .09 | -0.20 | 49.8 (8.9) | 50.7 (8.9) | -0.9 (-2.2, 0.5) | .15 | -0.17 |
| Fatigue | 46.6 (8.4) | 46.1 (8.4) | 0.5 (-0.9, 1.9) | .43 | 0.10 | 48.2 (8.2) | 46.9 (8.2) | 1.3 (-0.1, 2.7) | .04 | 0.25 |
| **PCa-specific symptoms: EPIC ^1,3^** |  |  |  |  |  |  |  |  |  |  |
| Urinary | 85.1 (20.0) | 83.6 (20.0) | 1.5 (-3.3, 6.2) | .48 | 0.13 | 85.4 (19.2) | 87.8 (19.2) | -2.4 (-7.1, 2.4) | .26 | -0.20 |
| Bowel | 96.8 (14.7) | 92.4 (14.7) | 4.4 (0.2, 8.6) | .02 | 0.42 | 94.1 (14.1) | 97.4 (14.1) | -3.4 (-7.6, 0.9) | .07 | -0.32 |
| Sexual | 46.7 (42.1) | 44.8 (42.1) | 1.9 (-5.9, 9.6) | .59 | 0.11 | 60.0 (37.8) | 62.2 (37.8) | -2.2 (-9.9, 5.5) | .52 | -0.13 |
| Hormonal | 85.0 (25.0) | 83.6 (25.0) | 1.3 (-5.6, 8.2) | .66 | 0.08 | 81.0 (24.0) | 78.8 (24.0) | 2.2 (-4.7, 9.1) | .48 | 0.13 |
|  | **Patients** | | | | | **Partners** | | | | |
|  | **Mean (SD)** | |  |  |  | **Mean (SD)** | |  |  |  |
|  | **Time point 1** | **Time point**  **2** | **Difference**  **(95% CI^4^)** | ***P* value^5^** | **Effect size^6^** | **Time point 1** | **Time point 2** | **Difference**  **(95% CI^4^)** | ***P* value^5^** | **Effect size^6^** |
| **T4 vs. T3** | **T4** | **T3** |  |  |  | **T4** | **T3** |  |  |  |
| **Primary outcomes**  **QOL FACT-G^1^** |  |  |  |  |  |  |  |  |  |  |
| FACT-G total score | 89.3 (11.1) | 89.3 (11.1) | 0.1 (-1.7, 1.8) | .91 | 0.01 | 85.9 (10.7) | 88.3 (10.7) | -2.4 (-4.1, -0.7) | .002 | -0.38 |
| **Secondary outcomes:** |  |  |  |  |  |  |  |  |  |  |
| **QOL subdomains^1^** |  |  |  |  |  |  |  |  |  |  |
| Physical | 24.7 (3.6) | 24.5 (3.6) | 0.1 (-0.4, 0.7) | .56 | 0.07 | 23.5 (3.4) | 23.7 (3.4) | -0.2 (-0.8, 0.4) | .46 | -0.09 |
| Social | 21.8 (4.2) | 22.1 (4.2) | -0.4 (-1.0, 0.3) | .24 | -0.14 | 21.7 (4.0) | 22.5 (4.0) | -0.7 (-1.4, -0.0) | .02 | -0.29 |
| Emotional | 20.5 (3.3) | 20.5 (3.3) | -0.0 (-0.6, 0.6) | .97 | -0.00 | 19.9 (3.1) | 20.5 (3.1) | -0.6 (-1.1, 0.0) | .03 | -0.26 |
| Functional | 22.2 (4.5) | 22.1 (4.5) | 0.2 (-0.5, 0.9) | .53 | 0.08 | 20.6 (4.3) | 21.4 (4.3) | -0.9 (-1.6, -0.2) | .004 | -0.35 |
| **Secondary outcomes: Psychosocial outcomes** |  |  |  |  |  |  |  |  |  |  |
| **Appraisals^1^** |  |  |  |  |  |  |  |  |  |  |
| Appraisal of illness | 3.8 (0.6) | 3.8 (0.6) | 0.0 (-0.1, 0.1) | .53 | 0.08 | 3.8 (0.6) | 3.9 (0.6) | -0.1 (-0.2, -0.0) | .003 | -0.36 |
| **Coping resources^1^** |  |  |  |  |  |  |  |  |  |  |
| Cancer Self-Efficacy Scale | 77.6 (11.6) | 77.8 (11.6) | -0.3 (-2.3, 1.8) | .78 | -0.03 | 78.0 (11.2) | 78.6 (11.2) | -0.7 (-2.7, 1.4) | .46 | -0.09 |
| **Social support^1^** |  |  |  |  |  |  |  |  |  |  |
| Emotional support | 55.4 (7.1) | 55.5 (7.1) | -0.1 (-1.3, 1.1) | .79 | -0.03 | 53.0 (6.9) | 53.7 (6.9) | -0.7 (-1.9, 0.5) | .20 | -0.16 |
| Informational support | 56.1 (7.9) | 56.8 (7.9) | -0.6 (-2.1, 0.9) | .35 | -0.11 | 55.8 (7.6) | 57.2 (7.6) | -1.4 (-2.8, 0.1) | .03 | -0.26 |
| Instrumental support | 58.4 (7.0) | 58.6 (7.0) | -0.1 (-1.3, 1.0) | .78 | -0.03 | 55.7 (6.7) | 56.6 (6.7) | -0.9 (-2.1, 0.2) | .07 | -0.22 |
| **Secondary outcomes: Symptom outcomes** |  |  |  |  |  |  |  |  |  |  |
| **General symptoms**^2^ |  |  |  |  |  |  |  |  |  |  |
| Anxiety | 46.9 (8.5) | 47.0 (8.5) | -0.1 (-1.5, 1.3) | .88 | -0.02 | 47.4 (8.2) | 46.9 (8.2) | 0.5 (-0.9, 1.9) | .44 | 0.09 |
| Depression | 45.7 (7.7) | 46.4 (7.7) | -0.7 (-1.9, 0.5) | .18 | -0.16 | 46.9 (7.5) | 45.5 (7.5) | 1.4 (0.2, 2.6) | .007 | 0.32 |
| Pain | 48.0 (8.9) | 48.5 (8.9) | -0.6 (-2.0, 0.9) | .37 | -0.11 | 52.5 (8.7) | 51.4 (8.7) | 1.1 (-0.3, 2.5) | .09 | 0.21 |
| Sleep | 47.9 (9.2) | 48.1 (9.2) | -0.2 (-1.6, 1.1) | .69 | -0.05 | 49.8 (8.9) | 50.2 (8.9) | -0.3 (-1.7, 1.0) | .60 | -0.06 |
| Fatigue | 46.6 (8.4) | 47.1 (8.4) | -0.5 (-2.0, 0.9) | .40 | -0.10 | 48.2 (8.2) | 48.0 (8.2) | 0.2 (-1.2, 1.6) | .78 | 0.03 |
| **PCa-specific symptoms: EPIC ^1,3^** |  |  |  |  |  |  |  |  |  |  |
| Urinary | 85.1 (20.0) | 87.1 (20.0) | -2.0 (-6.7, 2.7) | .34 | -0.17 | 85.4 (19.2) | 88.8 (19.2) | -3.4 (-8.1, 1.3) | .11 | -0.29 |
| Bowel | 96.8 (14.7) | 95.2 (14.7) | 1.6 (-2.6, 5.9) | .38 | 0.16 | 94.1 (14.1) | 96.4 (14.1) | -2.3 (-6.5, 1.9) | .22 | -0.22 |
| Sexual | 46.7 (42.1) | 44.0 (42.1) | 2.7 (-5.1, 10.4) | .44 | 0.15 | 60.0 (37.8) | 64.0 (37.8) | -3.9 (-11.7, 3.8) | .25 | -0.23 |
| Hormonal | 85.0 (25.0) | 84.9 (25.0) | 0.0 (-6.9, 6.9) | 1.00 | 0.00 | 81.0 (24.0) | 81.6 (24.0) | -0.6 (-7.5, 6.3) | .85 | -0.03 |

**Abbreviation:** QOL: quality of life; FACT-G: Functional Assessment of Chronic Illness Therapy-General; PCa: prostate cancer; EPIC, Expanded Prostate Cancer Index Composite; PERC: Prostate Cancer Education Resources for Couples.

**Footnote:**

1.Higher scores indicated more positive outcomes: ie, better quality of life, better perception of threat of symptoms, less severe symptoms, greater self-efficacy in symptom management, more social support, and better interpersonal support.

2.Higher scores indicated more negative outcomes: ie, more frequent or severe symptoms.

3.The EPIC-26 (26-item Expanded Prostate Cancer Index Composite) scores for patients and partners were standardized to enable direct comparison in subsequent analyses.

4. The 95% CIs represent Bonferroni-corrected simultaneous CIs for the mean differences between two groups, reported separately for patients and partners. CIs that do not include zero indicate statistically significant differences between groups.

5. The *P* values correspond to 2-sided tests of the null hypothesis that the mean difference between two groups equals zero. After applying Bonferroni correction for tests conducted separately in patients and partners, a *P* value less than 0.025 is considered statistically significant.

6. Effect sizes (Cohen *d*) are interpreted as small (0.2), medium (0.5), and large (0.8). Effects with |*d*|≥0.5 are considered potentially clinically meaningful.
