# Supplementary material for: Evaluating a Tailored Web-Based eHealth Intervention for Symptom Management in Couples Managing Prostate Cancer During the COVID-19 Pandemic: Randomized Clinical Trial
Source: J Med Internet Res. 2026 Jul 10;28:e88717. doi: 10.2196/88717 (PMC13358805; doi:10.2196/88717)
Supplement: Multimedia Appendix 6 [file jmir-v28-e88717-s006.docx]

|  | **Patients** | | | | | | | **Partners** | | | | | | | | | | |
| --- | --- | --- | --- | --- | --- | --- | --- | --- | --- | --- | --- | --- | --- | --- | --- | --- | --- | --- |
|  | **Mean (SD)** | | **Difference**  **(95% CI^4^)** | ***P* value^5^** | **Effect size^6^** | **Mean (SD)** | | | | | | **Difference**  **(95% CI^4^)** | | | ***P* value^5^** | **Effect size^6^** |  |  |
|  | **PERC** | **Control** |  |  |  | **PERC** | | | **Control** | | |  |  |  |  |  |  |  |
| **Time: T2** |  |  |  |  |  |  | | |  | | |  | | |  |  |  |  |
| **Primary outcomes**  **QOL FACT-G^1^** |  |  |  |  |  |  | | |  | | |  | | |  |  |  |  |
| FACT-G total score | 90.2 (10.1) | 89.4 (10.3) | 0.8 (-2.7, 4.3) | .61 | 0.09 | 89.7 (10.0) | | | 90.3 (9.9) | | | -0.6 (-4.1, 2.9) | | | .70 | -0.07 |  |  |
| **Secondary outcomes:** |  |  |  |  |  |  | | |  | | |  | | |  |  |  |  |
| **QOL subdomains^1^** |  |  |  |  |  |  | | |  | | |  | | |  |  |  |  |
| Physical | 24.4 (3.2) | 24.2 (3.3) | 0.3 (-0.9, 1.4) | .61 | 0.09 | 23.8 (3.2) | | | 23.8 (3.2) | | | 0.0 (-1.1, 1.1) | | | .99 | 0.00 |  |  |
| Social | 22.8 (3.8) | 22.2 (3.9) | 0.6 (-0.7, 1.9) | .29 | 0.18 | 23.5 (3.8) | | | 22.8 (3.7) | | | 0.7 (-0.6, 2.0) | | | .23 | 0.21 |  |  |
| Emotional | 20.7 (3.0) | 20.3 (3.0) | 0.4 (-0.7, 1.4) | .41 | 0.14 | 20.3 (3.0) | | | 20.5 (2.9) | | | -0.3 (-1.3, 0.8) | | | .56 | -0.10 |  |  |
| Functional | 22.0 (4.0) | 22.5 (4.1) | -0.5 (-1.9, 0.9) | .41 | -0.14 | 22.1 (4.0) | | | 22.9 (3.9) | | | -0.9 (-2.3, 0.5) | | | .17 | -0.23 |  |  |
| **Secondary outcomes: Psychosocial outcomes** |  |  |  |  |  |  | | |  | | |  | | |  |  |  |  |
| **Appraisals^1^** |  |  |  |  |  |  | | |  | | |  | | |  |  |  |  |
| Appraisal of illness | 3.9 (0.6) | 3.8 (0.6) | 0.1 (-0.1, 0.3) | .30 | 0.18 | 3.9 (0.6) | | | 3.9 (0.5) | | | 0.0 (-0.2, 0.2) | | | .90 | 0.02 |  |  |
| **Coping resources^1^** |  |  |  |  |  |  | | |  | | |  | | |  |  |  |  |
| Cancer Self-Efficacy Scale | 78.9 (10.6) | 79.9 (10.8) | -1.0 (-4.7, 2.7) | .54 | -0.10 | 79.2 (10.6) | | | 78.9 (10.3) | | | 0.3 (-3.4, 4.0) | | | .87 | 0.03 |  |  |
| **Social support^1^** |  |  |  |  |  |  | | |  | | |  | | |  |  |  |  |
| Emotional support | 55.6 (6.5) | 54.8 (6.6) | 0.8 (-1.5, 3.1) | .44 | 0.13 | 54.3 (6.5) | | | 54.3 (6.3) | | | 0.0 (-2.2, 2.3) | | | .98 | 0.00 |  |  |
| Informational support | 55.8 (7.2) | 55.7 (7.4) | 0.1 (-2.4, 2.7) | .90 | 0.02 | 56.9 (7.2) | | | 55.4 (7.1) | | | 1.4 (-1.1, 4.0) | | | .21 | 0.21 |  |  |
| Instrumental support | 58.7 (6.3) | 58.1 (6.5) | 0.6 (-1.6, 2.8) | .52 | 0.11 | 57.7 (6.3) | | | 56.3 (6.2) | | | 1.4 (-0.8, 3.6) | | | .14 | 0.25 |  |  |
| **Secondary outcomes: Symptom outcomes** |  |  |  |  |  |  | | |  | | |  | | |  |  |  |  |
| **General symptoms**^2^ |  |  |  |  |  |  | | |  | | |  | | |  |  |  |  |
| Anxiety | 47.3 (7.7) | 46.6 (7.9) | 0.7 (-2.0, 3.4) | .57 | 0.10 | 47.5 (7.7) | | | 45.7 (7.6) | | | 1.8 (-0.9, 4.5) | | | .14 | 0.25 |  |  |
| Depression | 46.3 (7.0) | 45.6 (7.2) | 0.6 (-1.8, 3.1) | .56 | 0.10 | 46.0 (7.0) | | | 44.9 (6.9) | | | 1.1 (-1.4, 3.5) | | | .33 | 0.17 |  |  |
| Pain | 48.2 (8.1) | 47.8 (8.3) | 0.4 (-2.4, 3.3) | .72 | 0.06 | 50.0 (8.1) | | | 50.5 (8.0) | | | -0.6 (-3.4, 2.3) | | | .66 | -0.08 |  |  |
| Sleep | 48.5 (8.3) | 49.3 (8.5) | -0.8 (-3.6, 2.1) | .55 | -0.10 | 50.3 (8.3) | | | 51.1 (8.1) | | | -0.8 (-3.6, 2.1) | | | .54 | -0.10 |  |  |
| Fatigue | 45.6 (7.7) | 46.6 (7.8) | -1.0 (-3.7, 1.6) | .38 | -0.15 | 46.9 (7.7) | | | 46.8 (7.6) | | | 0.1 (-2.6, 2.8) | | | .91 | 0.02 |  |  |
| **PCa-specific symptoms: EPIC ^1,3^** |  |  |  |  |  |  | | |  | | |  | | |  |  |  |  |
| Urinary | 85.2 (18.0) | 82.0 (18.5) | 3.2 (-6.4, 12.8) | .45 | 0.19 | 87.6 (17.6) | | | 88.0 (18.2) | | | -0.4 (-10.0, 9.2) | | | .93 | -0.02 |  |  |
| Bowel | 94.6 (13.4) | 90.2 (13.8) | 4.4 (-2.8, 11.6) | .17 | 0.35 | 95.1 (13.2) | | | 99.8 (13.4) | | | -4.7 (-12.0, 2.5) | | | .14 | -0.38 |  |  |
| Sexual | 47.1 (34.0) | 42.5 (35.7) | 4.6 (-11.7, 20.9) | .52 | 0.18 | 62.8 (31.9) | | | 61.7 (32.8) | | | 1.1 (-15.3, 17.4) | | | .88 | 0.04 |  |  |
| Hormonal | 82.4 (22.9) | 84.8 (23.3) | -2.4 (-14.5, 9.6) | .65 | -0.12 | 82.2 (22.5) | | | 75.4 (22.6) | | | 6.8 (-5.3, 18.8) | | | .21 | 0.32 |  |  |
|  | **Patients** | | | | | | | **Partners** | | | | | | | | | | |
|  | **Mean (SD)** | | **Difference**  **(95% CI^4^)** | ***P* value^5^** | **Effect size^6^** | | **Mean (SD)** | | | | **Difference**  **(95% CI^4^)** | | ***P* value^5^** | | | **Effect size^6^** |  |  |
|  | **PERC** | **Control** |  |  |  |  | **PERC** | | | **Control** |  |  |  |  |  |  | |  |
| **Time: T3** |  |  |  |  |  | |  | | |  |  | |  | | |  | |  |
| **Primary outcomes**  **QOL^1^ FACT-G** |  |  |  |  |  | |  | | |  |  | |  | | |  | |  |
| FACT-G total score | 90.8 (10.1) | 87.7 (10.3) | 3.0 (-0.5, 6.5) | .06 | 0.33 | | 88.3 (10.0) | | | 88.2 (9.9) | 0.1 (-3.4, 3.6) | | .94 | | | 0.01 | |  |
| **Secondary outcomes:** |  |  |  |  |  | |  | | |  |  | |  | | |  | |  |
| **QOL subdomains** |  |  |  |  |  | |  | | |  |  | |  | | |  | |  |
| Physical | 25.0 (3.2) | 24.0 (3.3) | 0.9 (-0.2, 2.1) | .06 | 0.32 | | 23.6 (3.2) | | | 23.7 (3.2) | -0.1 (-1.2, 1.0) | | .86 | | | -0.03 | |  |
| Social | 22.4 (3.8) | 21.8 (3.9) | 0.6 (-0.7, 1.9) | .30 | 0.18 | | 22.8 (3.8) | | | 22.1 (3.7) | 0.7 (-0.6, 2.0) | | .25 | | | 0.20 | |  |
| Emotional | 20.7 (3.0) | 20.4 (3.0) | 0.3 (-0.7, 1.4) | .49 | 0.12 | | 20.3 (3.0) | | | 20.7 (2.9) | -0.4 (-1.4, 0.7) | | .40 | | | -0.14 | |  |
| Functional | 22.5 (4.0) | 21.6 (4.1) | 0.9 (-0.5, 2.3) | .16 | 0.24 | | 21.5 (4.0) | | | 21.4 (3.9) | 0.1 (-1.2, 1.5) | | .81 | | | 0.04 | |  |
| **Secondary outcomes: Psychosocial outcomes** |  |  |  |  |  | |  | | |  |  | |  | | |  | |  |
| **Appraisals^1^** |  |  |  |  |  | |  | | |  |  | |  | | |  | |  |
| Appraisal of illness | 3.8 (0.6) | 3.8 (0.6) | 0.1 (-0.1, 0.2) | .55 | 0.10 | | 3.9 (0.6) | | | 3.9 (0.5) | -0.0 (-0.2, 0.2) | | .80 | | | -0.04 | |  |
| **Coping resources^1^** |  |  |  |  |  | |  | | |  |  | |  | | |  | |  |
| Cancer Self-Efficacy Scale | 78.1 (10.6) | 77.5 (10.8) | 0.7 (-3.0, 4.4) | .68 | 0.07 | | 77.9 (10.6) | | | 79.4 (10.3) | -1.4 (-5.2, 2.3) | | .38 | | | -0.15 | |  |
| **Social support^1^** |  |  |  |  |  | |  | | |  |  | |  | | |  | |  |
| Emotional support | 56.1 (6.5) | 55.0 (6.6) | 1.1 (-1.1, 3.4) | .27 | 0.19 | | 53.1 (6.5) | | | 54.3 (6.3) | -1.2 (-3.5, 1.0) | | .23 | | | -0.21 | |  |
| Informational support | 56.9 (7.2) | 56.6 (7.4) | 0.3 (-2.2, 2.9) | .78 | 0.05 | | 57.3 (7.2) | | | 57.1 (7.1) | 0.2 (-2.4, 2.7) | | .87 | | | 0.03 | |  |
| Instrumental support | 58.4 (6.3) | 58.7 (6.5) | -0.2 (-2.4, 1.9) | .80 | -0.04 | | 56.1 (6.3) | | | 57.1 (6.2) | -1.0 (-3.2, 1.2) | | .30 | | | -0.18 | |  |
| **Secondary outcomes: Symptom outcomes** |  |  |  |  |  | |  | | |  |  | |  | | |  | |  |
| **General symptoms**^2^ |  |  |  |  |  | |  | | |  |  | |  | | |  | |  |
| Anxiety | 47.3 (7.7) | 46.7 (7.9) | 0.5 (-2.2, 3.2) | .67 | 0.07 | | 47.4 (7.7) | | | 46.4 (7.6) | 1.0 (-1.7, 3.7) | | .39 | | | 0.15 | |  |
| Depression | 46.6 (7.0) | 46.2 (7.2) | 0.4 (-2.0, 2.9) | .69 | 0.07 | | 46.3 (7.0) | | | 44.7 (6.9) | 1.6 (-0.8, 4.0) | | .14 | | | 0.25 | |  |
| Pain | 47.6 (8.1) | 49.5 (8.3) | -1.9 (-4.7, 0.9) | .14 | -0.25 | | 51.4 (8.1) | | | 51.3 (8.0) | 0.1 (-2.8, 2.9) | | .97 | | | 0.01 | |  |
| Sleep | 47.4 (8.3) | 48.9 (8.5) | -1.5 (-4.4, 1.4) | .24 | -0.20 | | 50.6 (8.3) | | | 49.7 (8.1) | 0.9 (-2.0, 3.7) | | .50 | | | 0.12 | |  |
| Fatigue | 46.3 (7.7) | 48.0 (7.8) | -1.7 (-4.4, 1.0) | .16 | -0.24 | | 47.8 (7.7) | | | 48.2 (7.6) | -0.4 (-3.1, 2.3) | | .73 | | | -0.06 | |  |
| **PCa-specific symptoms: EPIC ^1,3^** |  |  |  |  |  | |  | | |  |  | |  | | |  | |  |
| Urinary | 87.2 (18.0) | 87.0 (18.5) | 0.2 (-9.4, 9.8) | .96 | 0.01 | | 90.4 (17.6) | | | 87.3 (18.2) | 3.1 (-6.5, 12.7) | | .47 | | | 0.19 | |  |
| Bowel | 96.5 (13.4) | 93.9 (13.8) | 2.6 (-4.7, 9.8) | .42 | 0.20 | | 97.8 (13.2) | | | 94.9 (13.4) | 2.9 (-4.3, 10.1) | | .37 | | | 0.23 | |  |
| Sexual | 46.1 (34.0) | 41.9 (35.7) | 4.2 (-12.1, 20.5) | .56 | 0.16 | | 67.1 (31.9) | | | 60.8 (32.8) | 6.3 (-10.1, 22.7) | | .39 | | | 0.24 | |  |
| Hormonal | 84.9 (22.9) | 85.0 (23.3) | -0.1 (-12.1, 11.9) | .99 | -0.00 | | 82.2 (22.5) | | | 81.0 (22.6) | 1.2 (-10.9, 13.3) | | .82 | | | 0.06 | |  |
|  | **Patients** | | | | | | | **Partners** | | | | | | | | | | |
|  | **Mean (SD)** | | **Difference**  **(95% CI^4^)** | ***P* value^5^** | **Effect size^6^** | | **Mean (SD)** | | | | **Difference**  **(95% CI^4^)** | | | ***P* value^5^** | | **Effect size^6^** | | |
|  | **PERC** | **Control** |  |  |  |  | **PERC** | | | **Control** |  |  |  |  |  |  |  |  |
| **Time: T4** |  |  |  |  |  | |  | | |  |  | | |  | |  | | |
| **Primary outcomes**  **QOL^1^ FACT-G** |  |  |  |  |  | |  | | |  |  | | |  | |  | | |
| FACT-G total score | 91.4 (10.1) | 87.3 (10.3) | 4.2 (0.6, 7.7) | .008 | 0.46 | | 86.5 (10.0) | | | 85.3 (9.9) | 1.3 (-2.3, 4.8) | | | .42 | | 0.14 | | |
| **Secondary outcomes:** |  |  |  |  |  | |  | | |  |  | | |  | |  | | |
| **QOL subdomains** |  |  |  |  |  | |  | | |  |  | | |  | |  | | |
| Physical | 25.2 (3.2) | 24.1 (3.3) | 1.0 (-0.1, 2.2) | .04 | 0.35 | | 23.7 (3.2) | | | 23.2 (3.2) | 0.5 (-0.7, 1.6) | | | .35 | | 0.16 | | |
| Social | 22.2 (3.8) | 21.3 (3.9) | 0.9 (-0.4, 2.3) | .11 | 0.27 | | 21.9 (3.8) | | | 21.6 (3.7) | 0.3 (-1.0, 1.6) | | | .60 | | 0.09 | | |
| Emotional | 20.7 (3.0) | 20.3 (3.0) | 0.5 (-0.6, 1.5) | .31 | 0.17 | | 20.0 (3.0) | | | 19.9 (2.9) | 0.1 (-1.0, 1.1) | | | .90 | | 0.02 | | |
| Functional | 23.0 (4.0) | 21.5 (4.1) | 1.5 (0.1, 2.9) | .01 | 0.42 | | 20.8 (4.0) | | | 20.3 (3.9) | 0.6 (-0.8, 2.0) | | | .34 | | 0.16 | | |
| **Secondary outcomes: Psychosocial outcomes** |  |  |  |  |  | |  | | |  |  | | |  | |  | | |
| **Appraisals^1^** |  |  |  |  |  | |  | | |  |  | | |  | |  | | |
| Appraisal of illness | 4.0 (0.6) | 3.7 (0.6) | 0.3 (0.1, 0.5) | .002 | 0.54 | | 3.8 (0.6) | | | 3.8 (0.5) | 0.1 (-0.1, 0.3) | | | .45 | | 0.13 | | |
| **Coping resources^1^** |  |  |  |  |  | |  | | |  |  | | |  | |  | | |
| Cancer Self-Efficacy Scale | 77.9 (10.6) | 77.2 (10.8) | 0.8 (-2.9, 4.5) | .64 | 0.08 | | 78.8 (10.6) | | | 77.1 (10.3) | 1.7 (-2.0, 5.4) | | | .30 | | 0.18 | | |
| **Social support^1^** |  |  |  |  |  | |  | | |  |  | | |  | |  | | |
| Emotional support | 56.2 (6.5) | 54.6 (6.6) | 1.6 (-0.7, 3.9) | .12 | 0.27 | | 53.2 (6.5) | | | 52.9 (6.3) | 0.3 (-2.0, 2.6) | | | .75 | | 0.05 | | |
| Informational support | 56.1 (7.2) | 56.2 (7.4) | -0.1 (-2.7, 2.4) | .92 | -0.02 | | 56.2 (7.2) | | | 55.4 (7.1) | 0.8 (-1.7, 3.3) | | | .48 | | 0.12 | | |
| Instrumental support | 59.4 (6.3) | 57.4 (6.5) | 2.0 (-0.2, 4.2) | .04 | 0.34 | | 55.2 (6.3) | | | 56.1 (6.2) | -0.9 (-3.1, 1.3) | | | .36 | | -0.15 | | |
| **Secondary outcomes: Symptom outcomes** |  |  |  |  |  | |  | | |  |  | | |  | |  | | |
| **General symptoms**^2^ |  |  |  |  |  | |  | | |  |  | | |  | |  | | |
| Anxiety | 46.2 (7.7) | 47.6 (7.9) | -1.4 (-4.1, 1.3) | .24 | -0.20 | | 46.7 (7.7) | | | 48.1 (7.6) | -1.4 (-4.1, 1.3) | | | .25 | | -0.20 | | |
| Depression | 44.8 (7.0) | 46.6 (7.2) | -1.7 (-4.2, 0.7) | .11 | -0.27 | | 46.9 (7.0) | | | 47.0 (6.9) | -0.1 (-2.5, 2.3) | | | .93 | | -0.02 | | |
| Pain | 46.4 (8.1) | 49.5 (8.3) | -3.2 (-6.0, -0.4) | .01 | -0.43 | | 51.7 (8.1) | | | 53.2 (8.0) | -1.5 (-4.4, 1.3) | | | .22 | | -0.21 | | |
| Sleep | 46.4 (8.3) | 49.4 (8.5) | -3.0 (-5.9, -0.2) | .02 | -0.41 | | 49.6 (8.3) | | | 50.1 (8.1) | -0.5 (-3.4, 2.4) | | | .69 | | -0.07 | | |
| Fatigue | 44.7 (7.7) | 48.5 (7.8) | -3.9 (-6.5, -1.2) | .001 | -0.55 | | 47.8 (7.7) | | | 48.5 (7.6) | -0.7 (-3.4, 2.0) | | | .56 | | -0.10 | | |
| **PCa-specific symptoms: EPIC ^1,3^** |  |  |  |  |  | |  | | |  |  | | |  | |  | | |
| Urinary | 86.2 (18.0) | 83.9 (18.5) | 2.3 (-7.2, 11.9) | .58 | 0.14 | | 85.7 (17.6) | | | 85.1 (18.2) | 0.6 (-9.0, 10.3) | | | .88 | | 0.04 | | |
| Bowel | 98.9 (13.4) | 94.7 (13.8) | 4.2 (-3.0, 11.4) | .19 | 0.34 | | 93.2 (13.2) | | | 94.9 (13.4) | -1.7 (-9.0, 5.5) | | | .59 | | -0.14 | | |
| Sexual | 46.2 (34.0) | 47.1 (35.7) | -0.9 (-17.2, 15.4) | .90 | -0.03 | | 54.1 (31.9) | | | 66.0 (32.8) | -11.9 (-28.3, 4.4) | | | .10 | | -0.46 | | |
| Hormonal | 85.5 (22.9) | 84.4 (23.3) | 1.1 (-11.0, 13.1) | .84 | 0.05 | | 76.8 (22.5) | | | 85.1 (22.6) | -8.3 (-20.4, 3.8) | | | .12 | | -0.39 | | |

**Abbreviation:** QOL: quality of life; FACT-G: Functional Assessment of Chronic Illness Therapy-General; PCa: prostate cancer; EPIC, Expanded Prostate Cancer Index Composite; PERC: Prostate Cancer Education Resources for Couples.

**Footnote:**

1.Higher scores indicated more positive outcomes: ie, better quality of life, better perception of threat of symptoms, less severe symptoms, greater self-efficacy in symptom management, more social support, and better interpersonal support.

2.Higher scores indicated more negative outcomes: ie, more frequent or severe symptoms.

3.The EPIC-26 (26-item Expanded Prostate Cancer Index Composite) scores for patients and partners were standardized to enable direct comparison in subsequent analyses.

4. The 95% CIs represent Bonferroni-corrected simultaneous CIs for the mean differences between two groups, reported separately for patients and partners. CIs that do not include zero indicate statistically significant differences between groups.

5. The *P* values correspond to 2-sided tests of the null hypothesis that the mean difference between two groups equals zero. After applying Bonferroni correction for tests conducted separately in patients and partners, a *P* value less than 0.025 is considered statistically significant.

6. Effect sizes (Cohen *d*) are interpreted as small (0.2), medium (0.5), and large (0.8). Effects with |*d*|≥0.5 are considered potentially clinically meaningful.
